# Phosphorylation of AHR by PLK1 promotes metastasis of LUAD via DIO2-TH signaling

**DOI:** 10.1371/journal.pgen.1011017

**Published:** 2023-11-21

**Authors:** Chaohao Li, Derek B. Allison, Daheng He, Fengyi Mao, Xinyi Wang, Piotr Rychahou, Ibrahim A. Imam, Yifan Kong, Qiongsi Zhang, Yanquan Zhang, Jinghui Liu, Ruixin Wang, Xiongjian Rao, Sai Wu, B. Mark Evers, Qing Shao, Chi Wang, Zhiguo Li, Xiaoqi Liu

**Affiliations:** 1 Department of Toxicology and Cancer Biology, University of Kentucky, Lexington, Kentucky, United States of America; 2 Department of Pathology and Laboratory Medicine, University of Kentucky, Lexington, Kentucky, United States of America; 3 Markey Cancer Center, University of Kentucky, Lexington, Kentucky, United States of America; 4 Department of Surgery, University of Kentucky, Lexington, Kentucky, United States of America; 5 Department of Chemical and Materials Engineering, University of Kentucky, Lexington, Kentucky, United States of America; 6 Department of Biostatistics, University of Kentucky, Lexington, Kentucky, United States of America; University Hospital Essen: Universitatsklinikum Essen, GERMANY

## Abstract

Metastasis of lung adenocarcinoma (LUAD) is a major cause of death in patients. Aryl hydrocarbon receptor (AHR), an important transcription factor, is involved in the initiation and progression of lung cancer. Polo-like kinase 1 (PLK1), a serine/threonine kinase, acts as an oncogene promoting the malignancy of multiple cancer types. However, the interaction between these two factors and their significance in lung cancer remain to be determined. In this study, we demonstrate that PLK1 phosphorylates AHR at S489 in LUAD, leading to epithelial-mesenchymal transition (EMT) and metastatic events. RNA-seq analyses reveal that type 2 deiodinase (DIO2) is responsible for EMT and enhanced metastatic potential. DIO2 converts tetraiodothyronine (T4) to triiodothyronine (T3), activating thyroid hormone (TH) signaling. In vitro and in vivo experiments demonstrate that treatment with T3 or T4 promotes the metastasis of LUAD, whereas depletion of DIO2 or a deiodinase inhibitor disrupts this property. Taking together, our results identify the AHR phosphorylation by PLK1 and subsequent activation of DIO2-TH signaling as mechanisms leading to LUAD metastasis. These findings can inform possible therapeutic interventions for this event.

## Author summary

The spread of lung cancer cells to other parts of the body is a major reason why people with this cancer often do not survive. We studied two important proteins in lung cancer: aryl hydrocarbon receptor (AHR) and Polo-like kinase 1 (PLK1), which are known to worsen many types of cancer. Our research found that PLK1 interacted with AHR in a process called phosphorylation. This interaction initiated a process called epithelial-mesenchymal transition (EMT) in cancer cells, helping them spread to other parts of the body. We also discovered that another molecule called type 2 deiodinase (DIO2) was responsible for this process. DIO2 converted one type of thyroid hormone (T4) into another (T3), which then sent signals that aided the spread of cancer cells. When we exposed lung cancer cells to more T3 or T4, they spread more. However, when we inhibited DIO2, the cancer cells could not spread as much. Our research demonstrated that the interaction between AHR and PLK1, the role of DIO2, and thyroid hormones were significant factors in lung cancer cells spread. Understanding this could help us discover new ways to stop or slow down this undesired process.

## Introduction

Lung cancer is the leading cause of cancer-related deaths in the US and accounts for one-fifth of the total cancer mortalities among all populations, with approximately 350 deaths each day according to the latest cancer statistics [1]. The initiation and progression of lung cancer is a multifaceted process characterized by intricate interactions and may be influenced by a multitude of risk factors [2]. Given the large number of patients and relatively low survival rate, lung cancer continues to impose a significant and demanding burden on our society and the healthcare system. Histologically, lung cancer is typically classified into two main categories: non-small cell lung cancer (NSCLC) and small cell lung cancer (SCLC). Of these, NSCLC accounts for over 80% of all cases and can be further differentiated into three primary subtypes: lung adenocarcinoma (LUAD), lung squamous cell carcinoma (LUSC), and large cell carcinoma [3]. Genetic mutations are the drivers of lung cancer, and drugs targeting these vulnerabilities are major treatments for lung cancer patients. Although great achievements have been made in developing targeted therapies for lung cancer, current treatment options fail to control disease progression in the long run owing to the emergence of resistance [4]. Therefore, a deeper investigation of the lung cancer etiology is necessary to better manage this disease.

Polo-like kinase 1 (PLK1) is a serine/threonine (S/T) protein kinase involved in the modulation of cell division at multiple phases, including mitotic entry, assembly of the kinetochore and spindle, centrosome maturation, activity of the anaphase-promoting complex, and cytokinesis [5–10]. Beyond its canonical role as a cell cycle regulator, PLK1 also possesses non-mitotic functions in other biological events, such as regulation of DNA damage repair, metabolism, and immune response [11–13]. Because of its wide functions, PLK1 is an oncogene frequently dysregulated in many cancers [14]. Specifically in NSCLC, PLK1 overexpression promotes lung cancer progression and is associated with poor patients’ outcomes [15,16]. Considering its importance in lung cancer, novel therapies targeting PLK1 have been developed with observable efficacy in lung cancer cells and animal models [17,18]. These preclinical findings are exceptionally promising, and further studies of PLK1 could significantly fortify the groundwork for advancing PLK1-based therapies in lung cancer patients’ treatment.

The aryl hydrocarbon receptor (AHR) is a ligand-activated transcription factor and a well-known sensor for xenobiotics [19]. Originally discovered as the receptor for the environmental toxicants that results in the carcinogenesis of liver and skin cancers, it has been found that AHR is associated with multiple stages of tumorigenesis [20]. Upon binding to various ligands, AHR dimerizes with cofactors and translocates to the nucleus, where it initiates the transcription of downstream targets that are important for tumor initiation, proliferation, and metastasis. AHR has been shown to be overexpressed in many NSCLC cell lines and patients [21,22]. In addition, high AHR activity is associated with poor overall survival and acquired resistance to treatment, suggesting its critical role as an oncogene in this deadly disease [23]. Therefore, AHR inhibitors are being developed and tested in clinical trials for the treatment of several solid tumors [24]. Although both PLK1 and AHR are closely related to lung cancer tumorigenesis, whether they cooperate to accelerate tumor progression remains unknown. Here, we show that AHR is a direct substrate of PLK1 and that phosphorylation of AHR in LUAD promotes epithelial–mesenchymal transition (EMT) to enhance the metastatic potential of cancer cells. Mechanistically, AHR phosphorylation elevates the expression of type 2 deiodinase (DIO2) and activates thyroid hormone (TH) signaling. Overall, our findings reveal a novel mechanism linking two important tumor promoters in lung cancer and provide translational values for lung cancer interventions.

## Methods

### Ethics statement

All animal experiments used in this study were approved by the University of Kentucky Division of Laboratory Animal Resources.

### In vitro kinase assay

**Fig 1:**
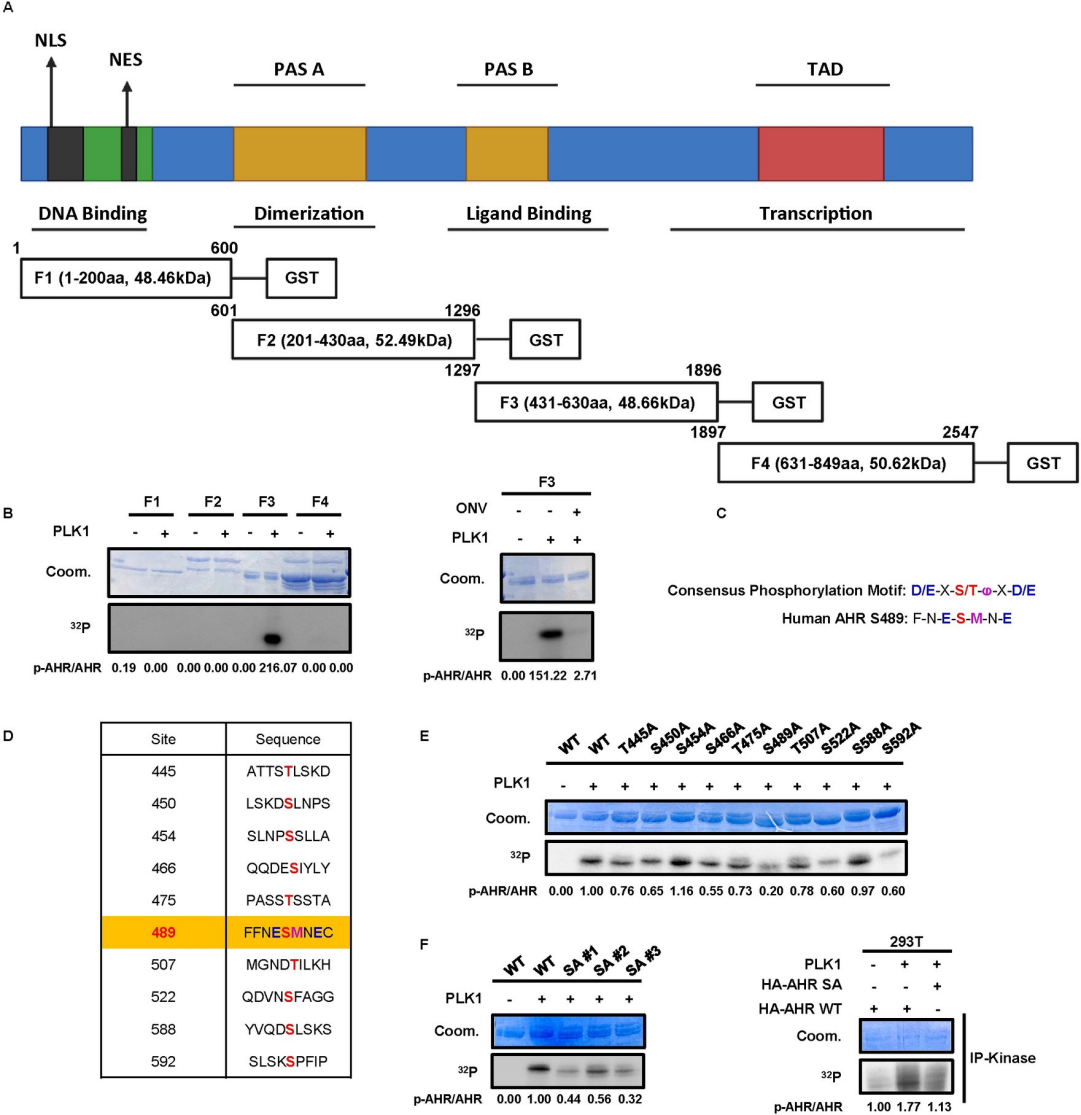
In vitro kinase assay identifies AHR as the PLK1 substrate. **A,** Fragmentation of full-length AHR ORF into F1, F2, F3, and F4 based on its functional domains. The C-termini of each fragment is tagged with glutathione s-transferase (GST). NLS, nuclear localization signal. NES, nuclear export signal. PAS, Per-Arnt-Sim domain. TAD, transactivation domain. Created with BioRender.com. **B,** In vitro kinase assay with four fragments. Coom, Coomassie Blue. ^32^P, radioactive phosphorus-32. The final concentration of ONV is 50nM. **C,** Previous identified consensus phosphorylation motif of PLK1 substrates and S489 site of F3. **D,** Potential PLK1 phosphorylation sites identified using the software. **E,** In vitro kinase assay with WT or mutated F3. **F,** In vitro kinase assay with WT and SA mutated F3 or HA-AHR.

**Fig 2:**
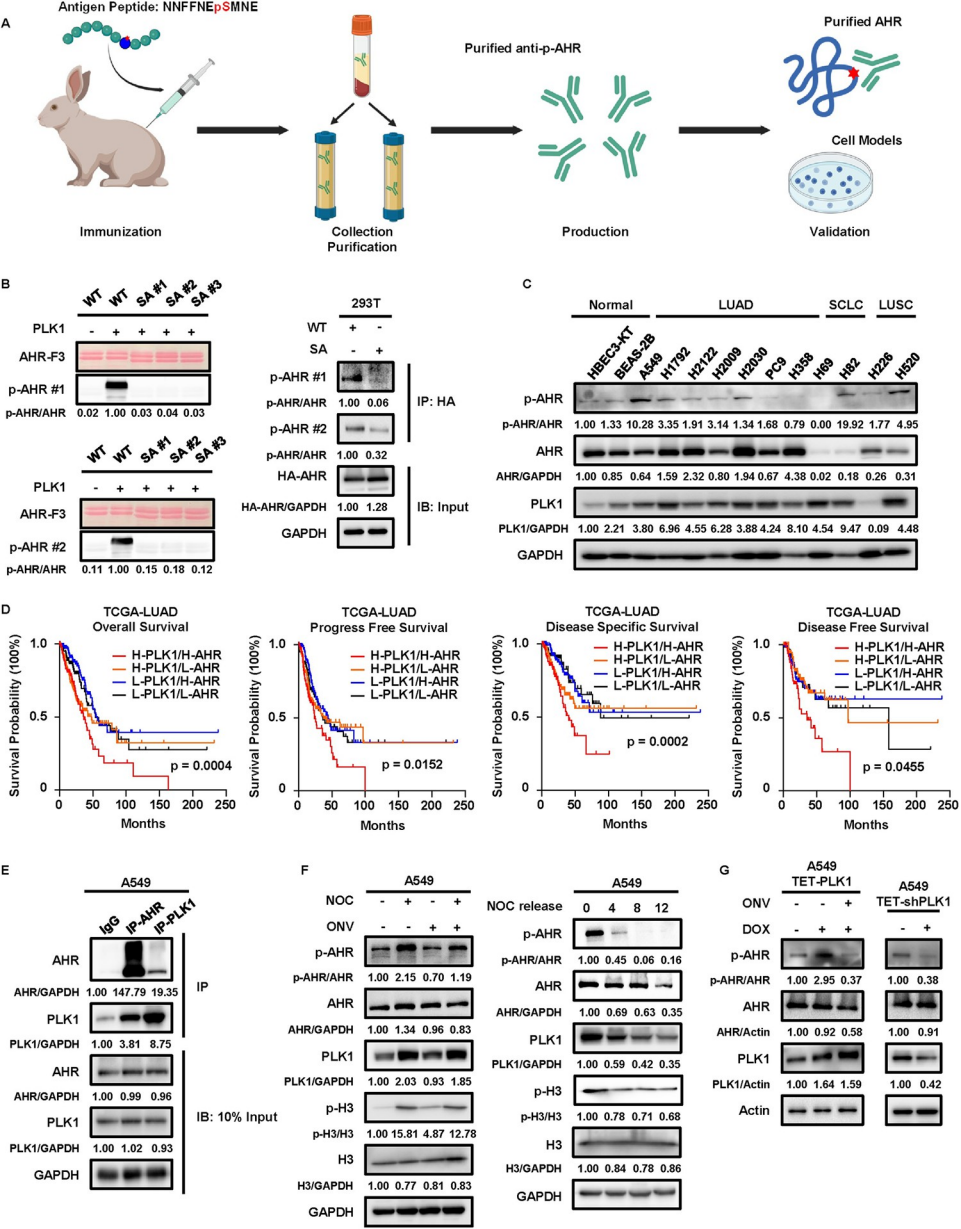
Interaction of AHR and PLK1 is implicated in LUAD progression. **A,** Illustration of p-AHR antibodies and validation experiments. Created with BioRender.com. **B,** Specificities validation of two anti-p-AHR antibodies with F3 or HA-AHR. **C,** IB to detect p-AHR in normal lung cells and different subtypes of lung cancers. **D,** Kaplan-Meier survival curves of TCGA-LUAD patients. Based on the median expressions of PLK1 and AHR, patients are separated into four groups: H-PLK1/H-AHR (PLK1/AHR > median), H-PLK1/L-AHR (PLK1 > median/AHR < median), L-PLK1/H-AHR (PLK1 < median/AHR > median), L-PLK1/L-AHR (PLK1/AHR < median). Statistical method: Log-rank test. **E,** Co-immunoprecipitation of AHR and PLK1 in A549 cells. 10% lysates are used as input control. **F,** IB to detect p-AHR after 18 hours NOC treatment, and NOC release experiments in A549 cells. The final concentrations used are 200ng/ml for NOC and 50nM for ONV. **G,** IB against p-AHR in TET-PLK1 and TET-shPLK1 A549 cells. Cells are treated with DOX for 48 hours to induce or deplete PLK1. The final concentrations used are 200ng/ml for DOX and 50nM for ONV.

Human AHR ORFs were cloned into the pGEX-KG vector. Protein purification of fragmented AHR was performed using the BL21 system and Glutathione Sepharose 4B (Cytiva, 17075601). Full-length human HA-AHR protein was purified using Pierce Anti-HA Magnetic Beads (Thermo, 88836) from 293T cell line transfected with HA-AHR plasmids. The detailed conditions of the in vitro kinase assay have been previously described [25]. Briefly, the reaction mixture was prepared with substrates, reaction buffers, and radioactive ATP and then incubated at 30°C for 30 mins, followed by SDS-PAGE gel electrophoresis. The gel was stained with 0.1% Coomassie Blue, destained, dried under vacuum, and subjected to autoradiography. Validation of phosphorylation antibodies by fragmented AHR proteins was performed using nonradioactive ATP, and 0.5% Ponceau S was used for gel staining. After destaining with 1x TBST buffer, the gel was subjected to immunoblotting. All kinase assays were repeated three times, except the kinase assays in the right panel of Fig 1F and the whole Fig 2B. One representative was shown.

### Prediction of phosphorylation and mutagenesis of plasmids

Prediction of phosphorylation sites by PLK1 was performed with GPS 5.0 and GPS-Polo 1.0 [26,27]. Mutagenesis of the HA-AHR plasmid was performed using Q5 Site-Directed Mutagenesis Kit (New England Biolabs, E0554S).

### Cell culture

The original A549 and PC9 cell lines were kindly provided by Dr. Qiou Wei at the University of Kentucky, and all other cell lines (293T, H1299 and cell lines in Fig 2C) were purchased from ATCC. Generation of V, WT, SA, and SD cell lines stably expressing different mutations of HA-AHR was performed by lentiviral overexpression of full-length HA-AHR ORFs or their empty vector, and 200ug/ml hygromycin was used to maintain cells. A549 TET-PLK1 and TET-shPLK1 cell lines were generated using the lentivirus packaged with TET-FLAG-PLK1 and TET-shPLK1 plasmids, and puromycin (2ug/ml) was used to maintain cells. Expressions of firefly luciferase and GFP were introduced using the lentivirus packaged with pCDH-EF1-Luc2-cG-BSD plasmid, and blasticidin (8ug/ml) was used to maintain cells. SD-shDIO2 cells (#1 and #2) were generated using the lentivirus packaged with two different shRNA plasmids and lentivirus expressing luciferase/GFP mentioned above. Puromycin (2ug/ml) and blasticidin (8ug/ml) were used to maintain cells. The HBEC3-KT cell line was cultured in Airway Epithelial Cell Basal Medium (ATCC, PCS-300-030) supplied with Bronchial Epithelial Cell Growth Kit (ATCC, PCS-300-040) at 37°C and 5% CO2. The BEAS-2B cell line was cultured in Bronchial Epithelial Cell Growth Basal Medium kit (Lonza, CC-3170) at 37°C and 5% CO2. The A549 and H1299 cell lines and their derivatives, as well as all other lung cancer cell lines were cultured in RPMI 1640 medium. 293T cells were cultured in high-glucose DMEM. The culture conditions for 293T and all the lung cancer cell lines were 10% FBS, 5% penicillin-streptomycin, 37°C, and 5% CO2. All the cell lines were within 50 passages and tested negative for mycoplasma contamination.

### Immunoblotting, immunoprecipitation and immunohistochemistry

**Immunoblotting**. Cell pellets were washed with ice-cold PBS and 1x RIPA buffers containing 50x protease inhibitor cocktail and 100x phosphatase inhibitor were used for lysis. Pierce BCA Protein Assay Kit (Thermo, 23225) was used to measure concentrations of lysates and 15μg total proteins were loaded onto SDS-PAGE gels for electrophoresis. After gel transfer to PVDF membranes, 5% skim milk was used to block the membranes. Primary antibodies in 1x TBST buffer were incubated overnight. After washing three times with 1x TBST buffer, HRP-linked secondary antibodies were applied in 1x TBST buffer for an hour, followed by another three washing with 1x TBST buffer. Bands were probed with SuperSignal West Dura Extended Duration Substrate (Thermo, 34076) and visualized with ChemiDoc Imaging System (Bio-Rad). All experiments were repeated three times (except for Figs 1F right, 2B, and S8F) and one representative was shown. Immunoblot replicates can be found in S2 Appendix. Immunoblots were analyzed and quantified by the Image Lab software (Bio-Rad). Results were normalized to one lane. **Immunoprecipitation**. Lysates containing 1mg total protein were incubated with anti-AHR, anti-PLK1 or IgG control antibodies overnight. The lysates were then incubated with PureProteome Protein A/G Mix Magnetic Beads (Sigma, LSKMAGAG10) at room temperature for 30 mins. After washing the beads with 1x TBST three times, samples were eluted by adding 60ul 1x SDS loading buffer and boiling at 90°C for 10 mins. The eluates were subject to regular immunoblotting. **Immunohistochemistry**. MCC-TMA slides were acquired from the University of Kentucky Markey Cancer Center. The staining of DIO2 was performed by Biospecimen Procurement And Translational Pathology Shared Resource Facility of Markey Cancer Center. Quality check and scoring of DIO2 was performed by pathologist Dr. Derek Allison.

### Wound healing assay

Cells were seeded in 6-well plates and cultured in complete medium until 100% confluence. 200ul tips were used to scratch the middle areas of the wells. Images were taken every 24 hours with the Nikon microscope and analyzed using Fiji [28]. Before taking pictures, cells were washed with 1x PBS twice to remove floating cells. Cells were cultured in medium with the indicated chemicals, and the concentration of FBS was 0.5%, which was optimized to avoid proliferation. All experiments were repeated three times and one representative picture was shown.

### Transwell assay

**Migration assay**. Cells (5 × 10^4^) were seeded on the top of 8.0 μm 24-well inserts (Corning, 353097) with 100ul medium containing 0.5% FBS and the indicated chemicals. 600ul complete medium were added to the bottom of each well to induce chemotaxis. After 24 hours, cells were fixed with 70% ethanol for 30 mins and stained with 0.1% crystal violet for 30 mins, then washed with tap water until clear. Cells on the top of the inserts were removed using cotton swabs. Images were taken with the Nikon microscope and analyzed using Fiji. **Invasion assay.** Matrigel (Corning, 354234) was diluted with medium containing 0.5% FBS at a ratio of 1:50. 50ul diluted mixture was used to coat the top of the inserts at 37°C for 30 mins. Subsequent procedures were the same as those used for the migration assay. All experiments were repeated three times and one representative picture was shown.

### 2D growth assay

Cells (2 × 10^3^ cells/well in 200μl) were seeded in 96-well plates overnight. On the second day (day 0), the medium was refreshed with the indicated concentrations of the chemicals. On day 3, AquaBluer (MultiTarget Pharmaceuticals LLC, 6015) reagent was mixed with the medium at a ratio of 1:100, the old medium was aspirated and 100ul mixture was added to each well, followed by incubation at 37°C for 4 hours. Fluorescence intensity at 540ex/590em was read by the GloMax Discover plate reader (Promega).

### 3D culture

**3D invasion assay.** Embedded 3D cultures were used in the assay. Briefly, Matrigel was diluted with medium containing 2% FBS at a ratio of 1:1, then 100ul diluted Matrigel was used to coat 24-well plates at 37°C for 30 mins. Next, the cell suspension (5 × 10^3^ cells/well) was mixed with diluted Matrigel at a ratio of 1:10, then 300ul mixture was added to the coated 24-well plates and incubated at 37°C for 30 mins. Finally, 500ul complete medium with the indicated chemicals was added to each well, and the culture was maintained for 6 to 8 days. The medium and chemicals were refreshed every two days. Images were taken with the Nikon microscope and analyzed using Fiji. **3D spheroid formation and growth assay**. The assay has been previously described with slight modifications [29]. Briefly, cells (1 × 10^3^ cells/well in 100μl) were diluted in 80% complete medium with 20% Matrigel, seeded onto 96-well plates and incubated at 37°C for 30 mins. Following Matrigel solidification, 50ul complete medium with indicated chemicals was added to each well and kept culture for 6 to 8 days. The medium and chemicals were refreshed every two days. On the last day, spheres were imaged with the Nikon microscope and analyzed using Fiji. Alternatively, AquaBluer reagent was mixed with the medium at a ratio of 1:100, the old medium was aspirated and 50ul mixture was added to each well, followed by incubation at 37°C for 4 hours. The fluorescence intensity at 540ex/590em was read by the GloMax Discover plate reader.

### Animal experiments

6-week-old athymic nude mice (The Jackson Lab, 002019) were used for all experiments. All tissues were formalin-fixed and paraffin-embedded (FFPE). Whole slides were scanned using the Aperio Digital Pathology Slide Scanner (Leica). Images were viewed and quantified using ImageScope (Leica). **Intravenous injection of V, WT, SA, and SD cells.** Cells (5 × 10^5^ in 100μl PBS) were intravenously injected into each nude mouse. All mice were euthanized at week 12 based on a previously reported study [30]. FFPE tissues were sent to the histological laboratory for hematoxylin and eosin (H&E) staining. **Intravenous injection of SD and SD-shDIO2 cells.** Iopanoic acid (IOP) was dissolved in 10% DMSO and 90% corn oil. SD and SD-shDIO2 (#1 and #2) cells (5 × 10^5^ in 100μl PBS) were intravenously injected into each nude mouse. Mice in the IOP treatment group were injected with SD cells and treated with 25mg/kg IOP daily via oral gavage. In vivo imaging of the lung metastasis was performed at week 11. All mice were euthanized at week 12 and FFPE tissues were sent to the histology laboratory for H&E staining. One mouse from SD-shDIO2 #1 group was sacrificed early due to deteriorating health condition and thus was not processed for later experiments. **Intracardiac injections of V, WT, SA, and SD cells.** Cells (1 × 10^6^ in 100μl PBS) were intracardially injected into each nude mouse. In vivo imaging of the lung metastasis was performed at week 4, when mice started to become moribund. All mice were euthanized immediately after the experiment. **Subcutaneous injections of V, WT, SA, and SD cells.** Cells (2 × 10^6^ in 100μl PBS) were subcutaneously inoculated into each mouse. The tumor sizes were measured weekly using a digital caliper. Tumor volumes were calculated using the formula: V = L × W^2^ × 0.52, where V is volume (mm^3^), L is length (mm), and W is width (mm). The treatment was stopped when the tumor volume reached 1000 mm^3^. At least 100ul whole blood of each mouse was collected from the heart of each mouse upon euthanasia and analyzed for GFP^+^ tumor cells. One mouse from V and SA groups failed blood collection and thus was not processed for detection of GFP^+^ tumor cells.

### Flow cytometry

All data were acquired on the BD Symphony A3 analyzer (BD Biosciences) and analyzed using FlowJo software (BD Biosciences). **Cell cycle analysis.** The cell cycle was analyzed using the Vybrant DyeCycle Violet Stain (Thermo, V35003). Briefly, Cells (1 × 10^6^) in 1ml complete media were stained with 1ul dye at 37°C for 30 mins. The fluorescence intensity was acquired at 405ex/440em**. Analysis of GFP**^**+**^ **tumor cells.** Blood was collected via terminal cardiac puncture of the right ventricle using a 23-G needle attached to a 3mL blood collection tube precoated with EDTA. Samples were subjected to 1x RBC Lysis Buffer (BioLegend, 420301) treatment, washed with PBS, and resuspended in 400ul PBS. Circulating tumor cells were collected at 488ex/510em.

### In vivo imaging of lung metastasis

Mice were intraperitoneally injected with 150mg/kg D-Luciferin (GoldBio, LUCK). 10 mins later, mice were anesthetized in chamber filled with isoflurane and subject to imaging on Lago X imaging system (Spectral Instruments Imaging) at 570 nm wavelength. The results were analyzed using Aura Imaging Software (Spectral Instruments Imaging).

### Statistical analysis

All results were analyzed with the statistical functions in GraphPad Prism 8 (GraphPad Software), except the results in S6D, which was analyzed by the package "lme4" in R-4.2.2. Normality and variance of results were checked, and appropriate statistical analyses were performed. Statistical significance was set at p values < 0.05. The detailed methods can be found in the corresponding figure legends.

Additional method information can be found in S1 Appendix.

## Results

### PLK1 phosphorylates AHR at S489

Preliminary screening of full-length human AHR ORF using software indicated a phosphorylation event by PLK1. To investigate this possibility, we cloned the full-length human AHR ORF sequence into four fragments (F1-F4) based on its functional domains (Fig 1A), and radioactive phosphorus-32 (^32^P) kinase assays were performed with purified F1-F4 proteins. The results showed that only F3 had a ^32^P signal, which disappeared upon treatment with the PLK1 inhibitor Onvansertib (ONV), suggesting that phosphorylation of AHR occurred in F3 (Fig 1B). A visual examination of F3 sequence revealed that the sequence around S489 was highly congruent with the previously identified consensus phosphorylation motif of PLK1 [31] (Fig 1C). Together with the potential sites predicted by the software (Fig 1D), we mutated each individual S/T to A in F3, and then performed ^32^P kinase assays with these mutated proteins to determine which mutation could lead to the reduction of ^32^P signal. Mutation of S489 to A (SA) resulted in the greatest reduction in ^32^P signal (Fig 1E). Additional ^32^P kinase assays with triplicate F3 proteins or full-length human AHR protein purified from 293T cells confirmed S489 as a bonafide phosphorylation site (Fig 1F). Notably, although this site was conserved among higher animals, the phosphorylation motif of PLK1 was not conserved between human and mice, suggesting that S489 was a unique PLK1 phosphorylation site in human (S1A Fig). Based on these results, we argued that human AHR was a substrate of PLK1.

### AHR phosphorylation is associated with LUAD progression

To confirm this phosphorylation event in cells, we ordered company-generated antibodies specifically targeting the phospho-S489 epitope of AHR (p-AHR) and verified their efficacy using purified AHR proteins or endogenous AHR in cell models (Fig 2A). The results showed that the two antibodies detected p-AHR in purified F3 proteins (wild-type and SA mutation) or full-length AHR proteins (wild-type and SA mutation) purified from 293T cells transfected with HA-AHR plasmids (Fig 2B). Conversely, these antibodies failed to detect p-AHR in two mice lung cancer cell lines (KP and KPP) that were established by us previously [16] (S1B Fig), confirming their specificity and further validating that the identified phosphorylation site was unique in human. Hence, we used the antibody with a lower background signal (p-AHR #1) in our experiments. Next, we detected p-AHR in different subtypes of human lung cell lines. The results showed that endogenous p-AHR could be detected in various lung cell lines (Fig 2C). Compared to normal lung cell lines, some cancer cell lines, such as the LUAD cell line A549, exhibited enhanced p-AHR levels. To further investigate the significance of AHR and PLK1 in lung cancer, we analyzed lung cell line expression data from DepMap (https://depmap.org/portal/) and compared their expressions of AHR and PLK1 (S2A Fig). Our analysis revealed higher PLK1 expression in lung cancer cell lines (LUAD, LUSC, SCLC) compared to normal lung cells, underscoring PLK1’s role as an oncogene. Additionally, LUAD and LUSC cell lines exhibited higher AHR expression compared to normal lung cell lines, although these results were statistically significant only in LUAD cell lines. Conversely, SCLC cell lines showed the lowest AHR expression compared to normal, LUAD, and LUSC cell lines. Besides, survival analysis based on a public SCLC database [32], showed no difference among patients with different expression levels of AHR and PLK1 (S2B Fig). The less abundant expression of AHR and lack of survival evidence in SCLC patients led to our less focus on this lung cancer subtype, and we then investigated the significance of AHR and PLK1 in other two lung cancer subtypes. Survival analysis of the TCGA-LUAD dataset revealed that patients with high PLK1 and high AHR had the worst outcomes regarding their four different survival results (Fig 2D and S1 Table). In contrast, this phenomenon was not observed in the TCGA-LUSC dataset (S2C Fig and S1 Table). We noted that the expressions of AHR and PLK1 showed different patterns in LUAD and LUSC patients (S2D Fig). LUAD patients had a higher level of AHR but a lower level of PLK1, and this discrepancy led to the different median cutoffs of patients’ classification in our survival analysis. To rule out the possibility that the disproportionate cutoffs might mask any survival differences, we performed a second survival analysis with absolute cutoffs, using the median expression of AHR in LUAD patients and the median expression of PLK1 in LUSC patients. After adjusting the thresholds, we observed significant worse outcomes in LUAD patients with high PLK1 and AHR regarding their three survivorships, but still no difference in LUSC patients (S2E Fig and S2 Table). Taking all together, we hypothesized that the interaction of AHR and PLK1 was important in LUAD and thus focused on this lung cancer subtype for our study. We used the A549 cell line, which displayed the highest p-AHR level, to dissect the functional significance of phosphorylation in LUAD. We first performed a co-immunoprecipitation experiment using A549 cell lines, and the results showed that endogenous AHR and PLK1 indeed bind with each other (Fig 2E), suggesting the kinase-substrate relationship. To confirm that the endogenous p-AHR was due to PLK1, we treated the A549 cell line with nocodazole (NOC), which could synchronize cells at M phase to elevate PLK1 level. The ratio of p-H3/H3 was used to validate the effect of NOC treatment, with a higher ratio indicating the effectiveness of NOC treatment. We observed an increased p-AHR after NOC treatment, which was reversed by PLK1 inhibitor ONV, and a NOC release experiment further supported this regulation, in which the removal of NOC decreased the PLK1 level that was accompanied by the decline of p-AHR (Fig 2F). To provide further evidence, we established tetracycline-controlled overexpression (TET-PLK1) and depletion (TET-shPLK1) of PLK1 systems in the A549 cell line. Treatment with doxycycline (DOX) to overexpress or deplete PLK1 witnessed an elevation or reduction of p-AHR (Fig 2G), confirming the phosphorylation of AHR by PLK1. Taking together, these data demonstrated that phosphorylation of AHR happened in LUAD and might be associated with disease status.

### Phosphorylation of AHR stimulates the EMT and metastatic potential of LUAD

**Fig 3:**
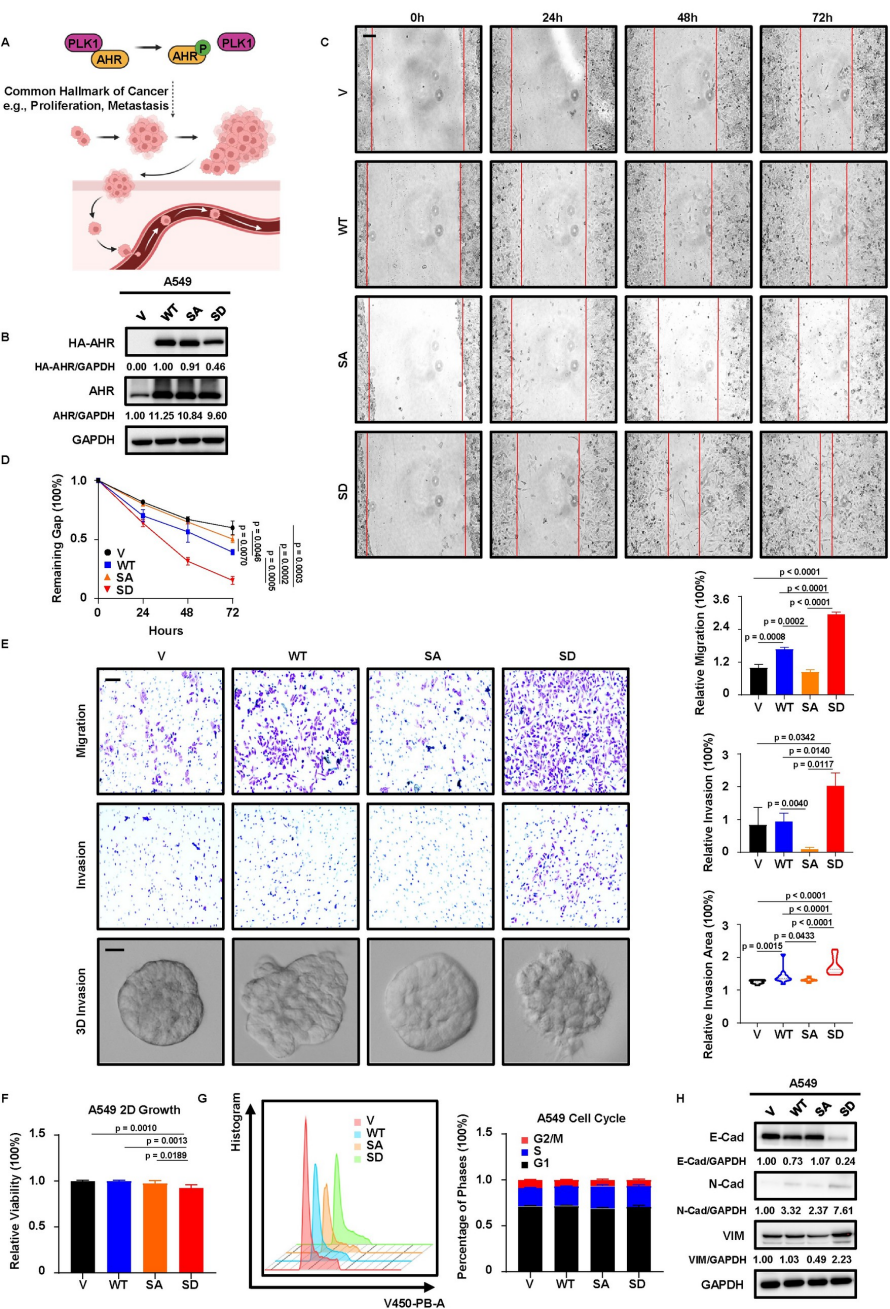
AHR phosphorylation promotes EMT and metastasis of LUAD. **A,** Possible outcomes of AHR phosphorylation by PLK1. Created with BioRender.com. **B,** IB to verify the establishment of A549 V, WT, SA, SD cells. **C, D,** Wound healing assay with V, WT, SA, SD cells. Results are normalized to 0h. Scale bar, 250μm. **E,** Transwell migration, invasion and 3D invasion assays with V, WT, SA, SD cells. Results are normalized to V and shown as mean ± SD (n = 3 for transwell migration and invasion assays, n = 10 for 3D invasion assay). Scale bar (upper two panels), 100μm. Scale bar (bottom panel), 20μm. Statistical methods: two-tailed unpaired Welch’s t test (invasion assay, SD-SA); two-tailed unpaired t test (remaining comparisons in transwell assays); two-tailed Mann-Whitney test (3D invasion assay). **F,** 3-day 2D growth assay with V, WT, SA, SD cells. Results are normalized to day 0 and shown as mean ± SD (n = 8). Statistical method: Welch’s ANOVA test following multiple comparisons. **G,** Cell cycle analysis of V, WT, SA, SD cells by flow cytometry. Results of each phase are scaled to 100% and shown as mean ± SD (n = 4). **H,** IB to detect EMT status and the associated E-Markers and M-Markers in V, WT, SA, SD cells.

To investigate the biological significance of p-AHR in LUAD, we intended to examine several hallmarks of cancer in LUAD (Fig 3A). By introducing an empty vector (V), wild-type AHR (WT, natural phosphorylation), AHR with SA mutation (0% phosphorylation), or AHR with a phosphomimetic mutation of S489 to D (SD, mimicking 100% phosphorylation) in A549 cell line (Fig 3B), we established four stable cell lines (hereafter referred to as V, WT, SA, SD) and used them to study the function of phosphorylation. We observed that WT and SD cells exhibited increased migratory abilities as demonstrated by the wound healing assay, with SD cells displaying the most significant enhancement (Fig 3C and 3D). This suggested that phosphorylation likely enhanced the metastatic potential of LUAD. Of note, PLK1 was previously reported to be a promoter of lung metastasis [15,33], and this was supported by our wound healing assays with TET-PLK1 and TET-shPLK1 systems (S3A Fig). We observed that DOX-induced overexpression of PLK1 led to the accelerated closure of scratches, whereas treatment with PLK1 inhibitor ONV could neutralize the acceleration of wound closure by DOX-induced overexpression. Conversely, DOX-induced depletion of PLK1 could decelerate wound closure. In addition, high AHR was also shown to be related to disease recurrence and distant metastasis in patients with LUAD [23]. Based on our validation experiments and literature results, we hypothesized that phosphorylation of AHR could promote the metastatic properties of LUAD. To test this hypothesis, we performed transwell migration and invasion assays. Besides, 3D invasion assay, which cultured cells in matrix-embedded medium, was also applied to provide a more realistic condition to mimic the in vivo environment, in which cancer cells had to cross the barriers of the extracellular matrix before the initiation of distant metastasis. Our results showed that, in comparison to V and SA cells, WT and SD cells demonstrated heightened abilities to penetrate the transwell membranes and the surrounding matrix, with SD cells consistently exhibiting the most significant difference (Fig 3E). These results supported the concept that AHR phosphorylation enhanced metastasis. To rule out the possibility that the observed phenotypes were due to the distinct proliferation rates, we checked the growth properties of V, WT, SA, and SD cells. Although the 2D growth assay indicated statistical significance between SD and other groups, the difference was minimal (less than 10%), and the slightly lower growth rate of SD cells alone could not account for their higher metastatic potential (Fig 3F). Additional experiments, including 3D spheroid formation, 3D growth assay, and cell cycle analysis, also failed to detect significant differences among four cell lines (Figs 3G, S3B and S3C). Apparently, the enhanced metastatic potential in WT and SD cells, especially the highest metastatic ability of SD cells, was not likely due to their differences in growth properties. These data were recapitulated in another LUAD cell line, H1299 (S4 Fig), further confirming the reliability of this phenomenon. Notably, PLK1 is an established inducer of EMT in multiple cancers [34], which is a precondition for metastatic events. To test whether this was responsible for the metastatic effect of phosphorylation, we detected some epithelial markers (E-Markers) and mesenchymal markers (M-Markers) in V, WT, SA, and SD cells. We observed that WT and SD cells exhibited elevated levels of M-Markers, N-Cadherin (N-Cad), and Vimentin (VIM), alone with reduced expression of the E-Marker, E-Cadherin (E-Cad). Particularly, SD cells displayed the highest levels of M-Markers and the lowest level of the E-Marker (Fig 3H), confirming the role of phosphorylation in promoting EMT. Taking together, these data supported the hypothesis that PLK1 phosphorylated AHR to enhance the metastatic potential of LUAD cells, and this phenotype might be due to the induction of EMT process.

### AHR phosphorylation promotes LUAD metastasis in vivo

**Fig 4:**
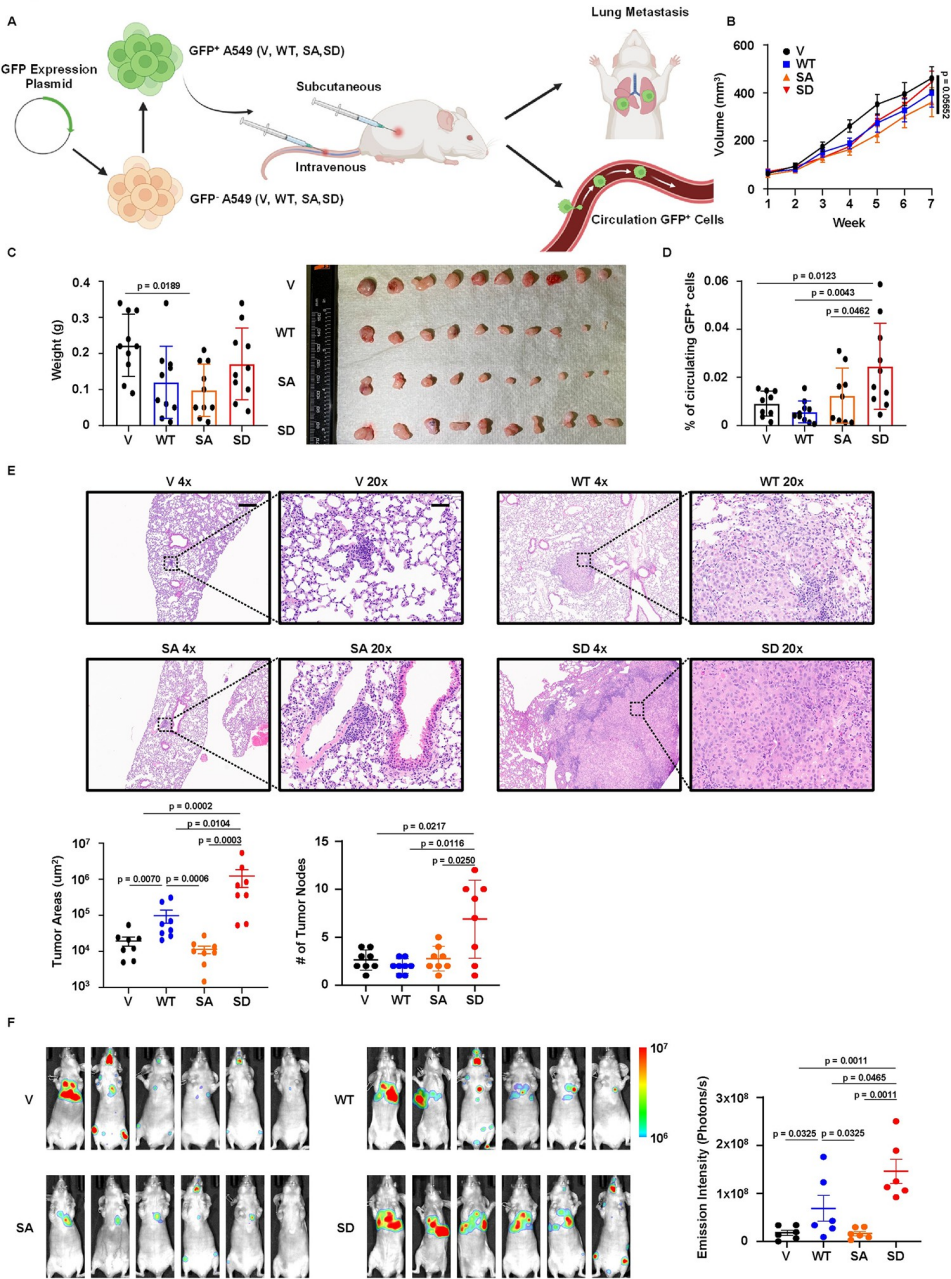
AHR phosphorylation promotes LUAD metastasis in vivo. **A,** Schematic representation of in vivo metastasis experiments. GFP^+^, GFP positive. GFP^-^, GFP negative. Created with BioRender.com. **B,** Tumor growth of V, WT, SA, SD cells over 7 weeks. Results are shown as mean ± SEM (n = 10). Statistical method: one-way ANOVA test. **C,** Final tumor weights and photograph of V, WT, SA, SD tumors. Results are shown as mean ± SD (n = 10). Statistical method, one-way ANOVA following multiple comparisons. **D,** Flow cytometry analysis of circulating GFP^+^ tumor cells. Results are shown as mean ± SD (n = 10 for WT, SD, and n = 9 for V, SA). Statistical method: one-tailed unpaired Welch’s t test. **E,** H&E staining of lung tissue sections of mice intravenously injected with V, WT, SA, SD cells. Scale bar (4x), 200μm. Scale bar (20x), 40μm. Results of quantification (n = 8) are shown as mean ± SEM for tumor areas and mean ± SD for tumor nodes. Statistical methods: two-tailed Mann-Whitney test (tumor areas); two-tailed unpaired Welch’s t test (tumor nodes). **F,** In vivo imaging of mice intracardially injected with V, WT, SA, SD cells. Results of quantification are shown as mean ± SEM (n = 6). Statistical method: one-tailed Mann-Whitney test.

Metastasis of primary tumors to distant tissues involves several critical steps, including local invasion, intravasation, survival in the circulatory system, extravasation, and residency at secondary sites [35]. To validate our in vitro findings regarding metastasis and to mimic this process, we introduced GFP and luciferase expressions in V, WT, SA, and SD cells, and designed two pioneering animal experiments to assess the metastatic abilities of these cells (Fig 4A). First, we subcutaneously inoculated V, WT, SA, and SD cells into nude mice, which mimicked the first two steps of metastasis. Tumor growth over 2 months was not statistically significance among V, WT, SA, and SD groups, however the final tumor weights displayed a slight difference between the V and SA cells (Fig 4B and 4C). Blood samples analysis from V, WT, SA, and SD groups revealed that mice inoculated with SD cells had a higher percentage of circulating GFP-positive cancer cells compared to other groups (Figs 4D and S5). This suggested a stronger tendency of SD cells to invade and intravasate into the circulatory system. Next, we intravenously injected V, WT, SA, and SD cell lines into nude mice and monitored the extent of lung residency, which mimicked the last three steps of metastasis. We found that WT and SD cells displayed larger lung metastatic lesions compared to V and SA cells, and SD cells exhibited both the largest metastatic lesions and the most frequent tumor nodes in lung tissues (Fig 4E). To capture the metastasis to the other sites, we performed intracardiac injection of V, WT, SA, and SD cells and detected the whole-body metastasis using in vivo bioluminescence (Fig 4F). In congruent with our previous results, WT and SD cells displayed stronger metastatic abilities compared to V and SA cells, with SD cells showing the strongest extent of metastasis. In summary, these data demonstrated that AHR phosphorylation stimulated metastasis of LUAD in vivo.

### Sequencing analysis identifies DIO2 as a molecular determinant of metastasis in LUAD

**Fig 5:**
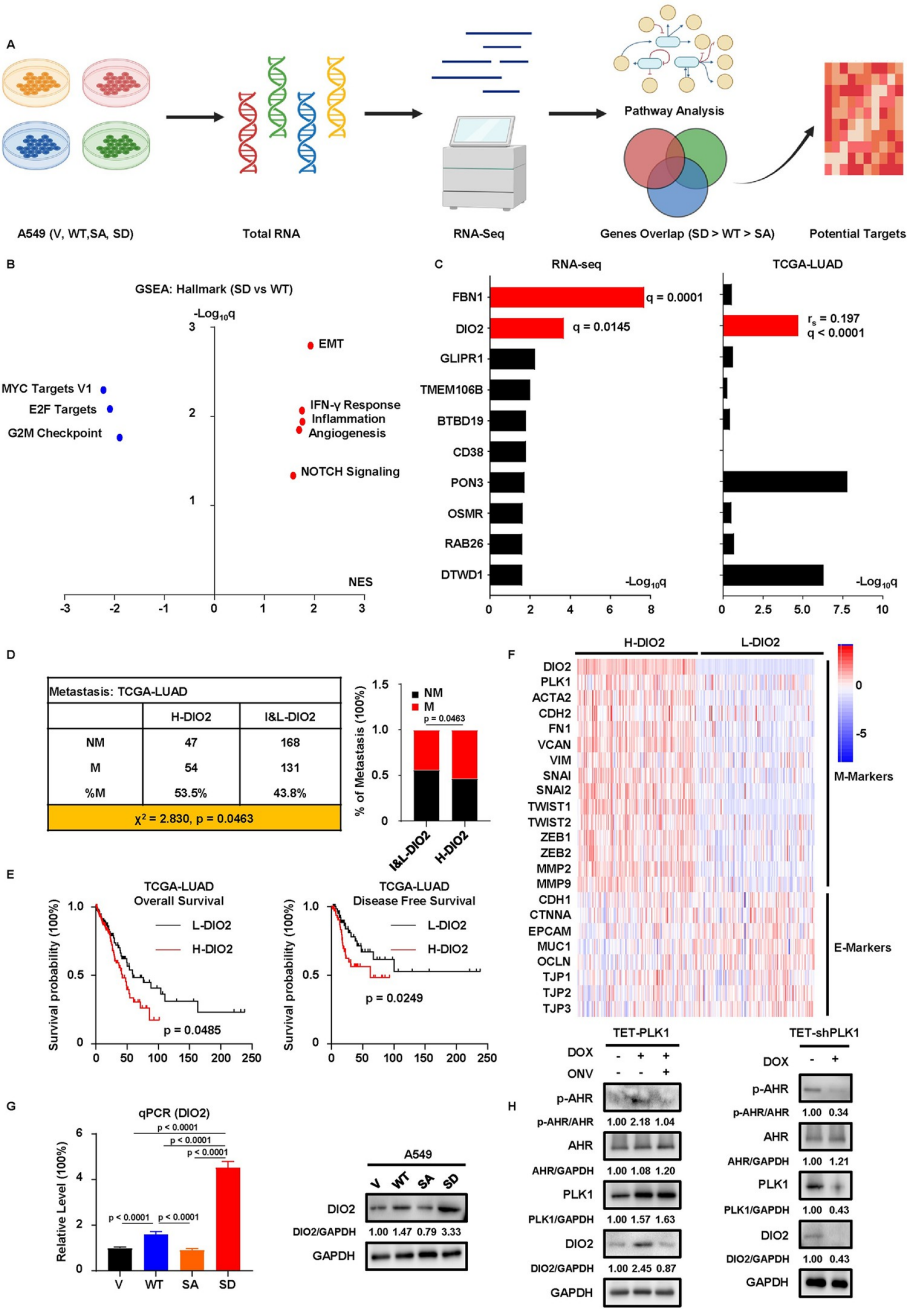
RNA-seq indicates DIO2 as a candidate responsible for metastasis. **A,** Simplified graphic representation of RNA-seq analysis. Created with BioRender.com. **B,** Dotplot (SD vs WT) of significantly upregulated (red color) and downregulated (blue color) pathways in SD cells (q values < 0.05), identified by GSEA with hallmark gene set. NES, normalized enrichment score. **C,** Barplots of top 10 upregulated genes (SD > WT > SA = V) identified from RNA-seq and their spearman correlations with PLK1 from TCGA-LUAD. Genes with q values (fold change of SD vs WT) < 0.05 in RNA-seq are marked with red colors at left panel. Genes with positive correlations and q values (Spearman r) < 0.05 in TCGA-LUAD are marked with red color at right panel. r_s_, Spearman correlation coefficient. **D,** Analysis of metastatic events of TCGA-LUAD patients. Based on the 3^rd^ quartile expression of DIO2, patients are separated into two groups: High DIO2 (H-DIO2, DIO2 > = 3^rd^ quartile) and Intermediate/Low DIO2 (I&L-DIO2, DIO2 < 3^rd^ quartile). Metastatic events are characterized by N > 0 or M1. NM, non-metastatic. M, metastatic. Statistical method: one-sided Chi-square test. **E,** Kaplan-Meier survival curves of TCGA-LUAD patients. Based on the 1^st^ and 3^rd^ quartile expression of DIO2, patients are separated into two groups: H-DIO2 (DIO2 > = 3^rd^ quartile) and L-DIO2 (DIO2 < = 1^st^ quartile). Statistical method: Log-rank test. **F,** Heatmap of EMT markers (z-scores of Log_2_TPM+1) in TCGA-LUAD patients. Based on the 1^st^ and 3^rd^ quartile expression of DIO2, patients are separated into two groups: H-DIO2 (DIO2 > = 3^rd^ quartile) and L-DIO2 (DIO2 < = 1^st^ quartile). **G,** qPCR and IB to detect the expression of DIO2 in V, WT, SA, SD cells. Results of qPCR are normalized to V and shown as mean ± SD (n = 8). Statistical methods: two-tailed unpaired t test (WT-SA, WT-SD); two-tailed unpaired Welch’s t test (WT-V, SD-V, SD-SA). **H,** IB to detect DIO2 in TET-PLK1 and TET-shPLK1 A549 cells. Cells are treated with DOX for 48 hours to induce or deplete PLK1. The final concentrations used are 200ng/ml for DOX and 50nM for ONV.

To investigate the mechanisms underlying AHR phosphorylation and metastasis, we performed RNA-seq using V, WT, SA, and SD cell lines (Fig 5A). Gene Set Enrichment Analysis (GSEA) with hallmark gene set showed that EMT was among the top-upregulated pathways in SD cells compared to WT or SA cells (Figs 5B, S6A, S6B and S3 Table), consistent with our previous findings regarding EMT markers expressions (Fig 3H). To further explore the molecular determinants contributing to the strongest metastatic potential in SD cells, we applied a stringent filter so that only genes expressing highest in SD cells, lowest in SA cells, and insignificant between SA and V cells (SD > WT > SA = V), were selected. We then ranked these genes according to their significance (q values of the SD vs WT fold changes) and would only focus on the genes that were significantly upregulated in SD cells (q values < 0.05). Besides, those genes should be positively correlated with PLK1, as we had the assumption that a higher PLK1 should result in a higher proportion of AHR phosphorylation and expression of its downstream targets. Based on this standard, we identified two statistically upregulated genes in SD cells from the top 10 gene list of RNA-seq, but only one of them, the type 2 deiodinase (DIO2), was positively correlated with PLK1 with a statistical significance (q values of the Spearman r < 0.05) in TCGA-LUAD dataset (Fig 5C and S4 Table), suggesting it as a candidate responsible for the metastasis phenotype. To validate the significance of DIO2, we further analyzed its relationship with PLK1 and disease outcomes in the TCGA-LUAD dataset. Initially, DIO2 showed a mild positive correlation with PLK1 in normal lung samples, but this correlation intensified significantly (smaller p value) in tumor samples (S6C Fig). Compared to normal lung tissues, both PLK1 and DIO2 levels were elevated in tumor samples and at higher stages of LUAD (S6D Fig). Of note, DIO2 was only statistically higher in LUAD of IIB stage when local metastasis started to appear, suggesting its importance during the early onset of metastasis. To investigate the correlation between DIO2 expression and survival, we separated patients into low DIO2 (L-DIO2), intermediate DIO2, and high DIO2 (H-DIO2) groups. Compared to the intermediate and L-DIO2 groups, the H-DIO2 group had a higher rate of metastatic events (Fig 5D). In addition, patients in the L-DIO2 group exhibited superior survival outcomes compared to those in the H-DIO2 group, displaying substantial improvements in overall survival and disease free survival (Figs 5E, S6E, S6F and S5 Table). Furthermore, H-DIO2 closely correlated with higher expressions of M-Markers and lower expressions of E-Markers (Figs 5F, S7A and S7B), consistent with our previous results that SD cells displayed a mesenchymal-like state favoring the initiation of metastasis (Fig 3H). Indeed, DIO2 was recently shown to promote the progression and invasiveness of skin cancer by inducing EMT [36]. However, this tumor-promoting property was not determined in LUAD. Based on these preconditions, we focused on DIO2 and argued that DIO2 was critical for the metastatic phenotype of SD cells. We verified the expression order of DIO2 by detecting the RNA and protein levels in V, WT, SA, and SD cell lines (Fig 5G). Furthermore, the modulation of PLK1 in the TET-FLAG and TET-shPLK1 systems confirmed the regulation of DIO2 by PLK1 (Fig 5H). Since AHR was a transcription factor, a reasonable guess could be that AHR phosphorylation elevated DIO2 via affecting AHR transcriptional activity. To test this possibility, we focused on the ligand-binding pocket (PAS-B domain) of AHR. A visual examination of the protein structures of PAS-B domains in WT and phospho-S489 AHR identified observable differences (S8A and S8B Fig). To characterize how these differences would shape the AHR activity, we performed two computational simulations of WT and phospho-S489 AHR proteins. Simulation of Radius of Gyration (RoG) showed lower RoG values in the PAS-B domain of phospho-S489 AHR (S8C Fig), indicating that atoms in the PAS-B domain of phospho-S489 AHR were close to their center of mass and thus a more compact structure. This feature suggested that the PAS-B domain of phospho-S489 AHR had less flexibility to accommodate its ligands. A further simulation of Solvent-Accessible Surface Area showed that the PAS-B domain of phospho-S489 AHR had fewer surface areas accessible to surrounding environment, suggesting a less likelihood to bind to its ligands (S8D Fig). These results indicated that AHR phosphorylation might lead to lower transcription activity, and this was validated by qPCR detecting AHR downstream targets in WT, SA, and SD cells, in which SA cells expressed more AHR downstream targets than WT cells but SD cells expressed less those targets than WT cells (S8E Fig). Given the reduced AHR activity in SD cells and the higher expression of DIO2, it was likely that DIO2 expression was negatively regulated by AHR activity. To test this assumption, we treated A549 cells with AHR inhibitor CH-223191 and did observe an increase of DIO2 (S8F Fig). These results provided mechanistic evidence linking AHR phosphorylation and DIO2 expression. Taking together, our results suggested that elevation of DIO2 upon AHR phosphorylation might be responsible for enhanced metastatic abilities in LUAD.

### DIO2 and TH enhance the metastatic capabilities of LUAD

**Fig 6:**
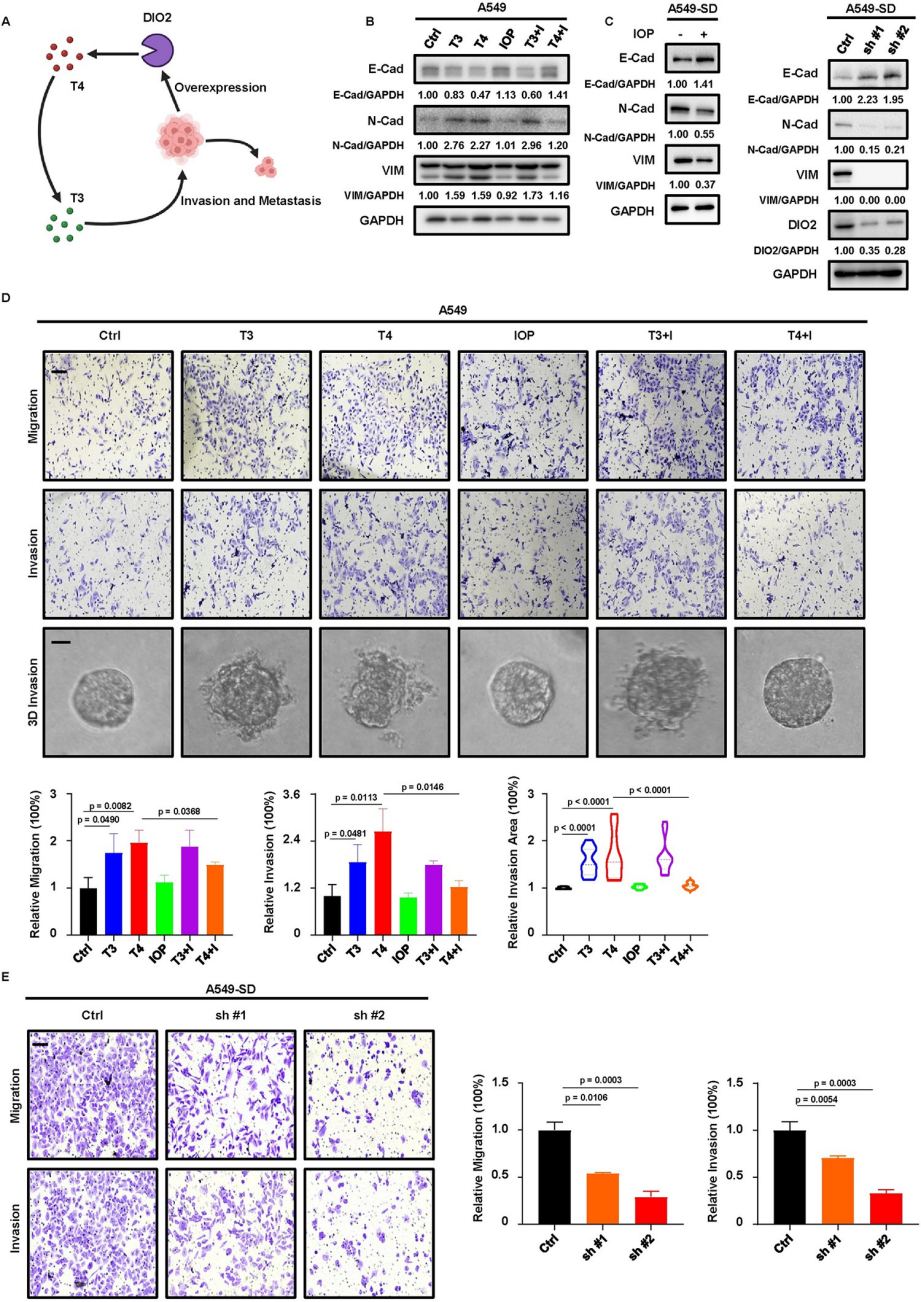
DIO2-TH signaling promotes metastasis of LUAD. **A,** Function of DIO2 in TH metabolism, represented by graph. Created with BioRender.com. **B,** IB to detect EMT markers in A549 cells treated with indicated chemicals for 48 hours. T3+I, T3+IOP. T4+I, T4+IOP. Untreated cells serve as control (Ctrl). The final concentrations used are 10nM for T3 and T4, and 50μM for IOP. **C,** IB to detect EMT markers in SD cells treated with 50μM IOP for 48 hours, or SD cells depleting DIO2 by shRNA (sh #1, sh #2). **D,** Transwell migration, invasion and 3D invasion assays with A549 cells treated with T3, T4, IOP, T3+I, T4+I. The final concentrations used are 10nM for T3 and T4, and 50μM for IOP. Results are normalized to Ctrl and shown as mean ± SD (n = 3 for transwell migration and invasion assays and n = 10 for 3D invasion assay). Scale bar (upper two panels), 100μm. Scale bar (bottom panel), 20μm. Statistical methods: two-tailed unpaired t test (transwell); two-tailed Mann-Whitney test (3D invasion). **E,** Transwell migration and invasion assays with SD cells depleting DIO2 by shRNA. Results are normalized to Ctrl and shown as mean ± SD (n = 3 for both assays). Scale bar, 100μm. Statistical methods: two-tailed unpaired Welch’s t test (migration, Ctrl-sh #1); two-tailed unpaired t test (remaining comparisons). **F,** 3D invasion assay with SD cells treated with IOP or depleting DIO2 by shRNA. Results are normalized to Ctrl and shown as mean ± SD (n = 10). Scale bar, 20μm. Statistical method: two-tailed Mann-Whitney test.

DIO2 is a key enzyme involved in metabolism of TH (Fig 6A). Two major forms of TH, tetraiodothyronine (T4) and triiodothyronine (T3), exist in the human body. DIO2 is mainly responsible for converting T4 to T3, and T3 is much more potent than T4 in activating TH signaling. Since we identified DIO2 as the candidate responsible for the enhanced metastasis after AHR phosphorylation, we hypothesized that DIO2-TH signaling promoted the EMT process and metastasis. To test this hypothesis, we started by treating A549 cells with TH to determine whether TH could influence metastatic abilities (Fig 6B). As expected, treatment with T3 or T4 elevated M-Markers and attenuated E-Markers in A459 cells. Addition of the DIO2 inhibitor IOP had a limited impact on T3 but disrupted the effect of T4, demonstrating the dependency of T4’s effect on DIO2 and emphasizing the importance of converting T4 to T3 by DIO2. Besides, treatment with IOP in SD cells, which had higher expression of DIO2, or depleting DIO2 by shRNA in SD cells led to elevation of E-Markers and reduction of M-Markers, confirming the positive regulation of DIO2 and TH on EMT process (Fig 6C). Next, we performed wound healing, transwell, and 3D invasion assays with A549 cells treated with TH (Figs 6D and S9A). We found that T3 and T4 enhanced the metastatic potential of A549 cells, and IOP treatment could only reverse T4’s effect but had no effect on T3, further confirming the reliance of T4’s effect on DIO2. Apparently, the enhanced metastasis driven by THs was not due to their impact on growth property, as we did not observe a significant change of A549 growth rate (S9B Fig). Additional experiments with siRNA-induced DIO2 knockdown in SD cells also supported the metastasis-promoting effect of DIO2, in which depletion of DIO2 mimicked the effect of IOP on inhibiting EMT and metastasis (S9C, S9D and S9E Fig). Finally, we also used shRNA to stably deplete DIO2 in SD cells, and this manipulation recapitulated the results of transient DIO2 depletion or IOP treatment, leading to suppression of metastasis without significantly affecting cell growth (Figs 6E, S9F, S9G and S9H). Taking together, these experiments demonstrated that DIO2-TH signaling accounted for the metastasis-promoting effect of AHR phosphorylation in LUAD.

### DIO2 and thyropathies are associated with LUAD patients’ outcomes

**Fig 7:**
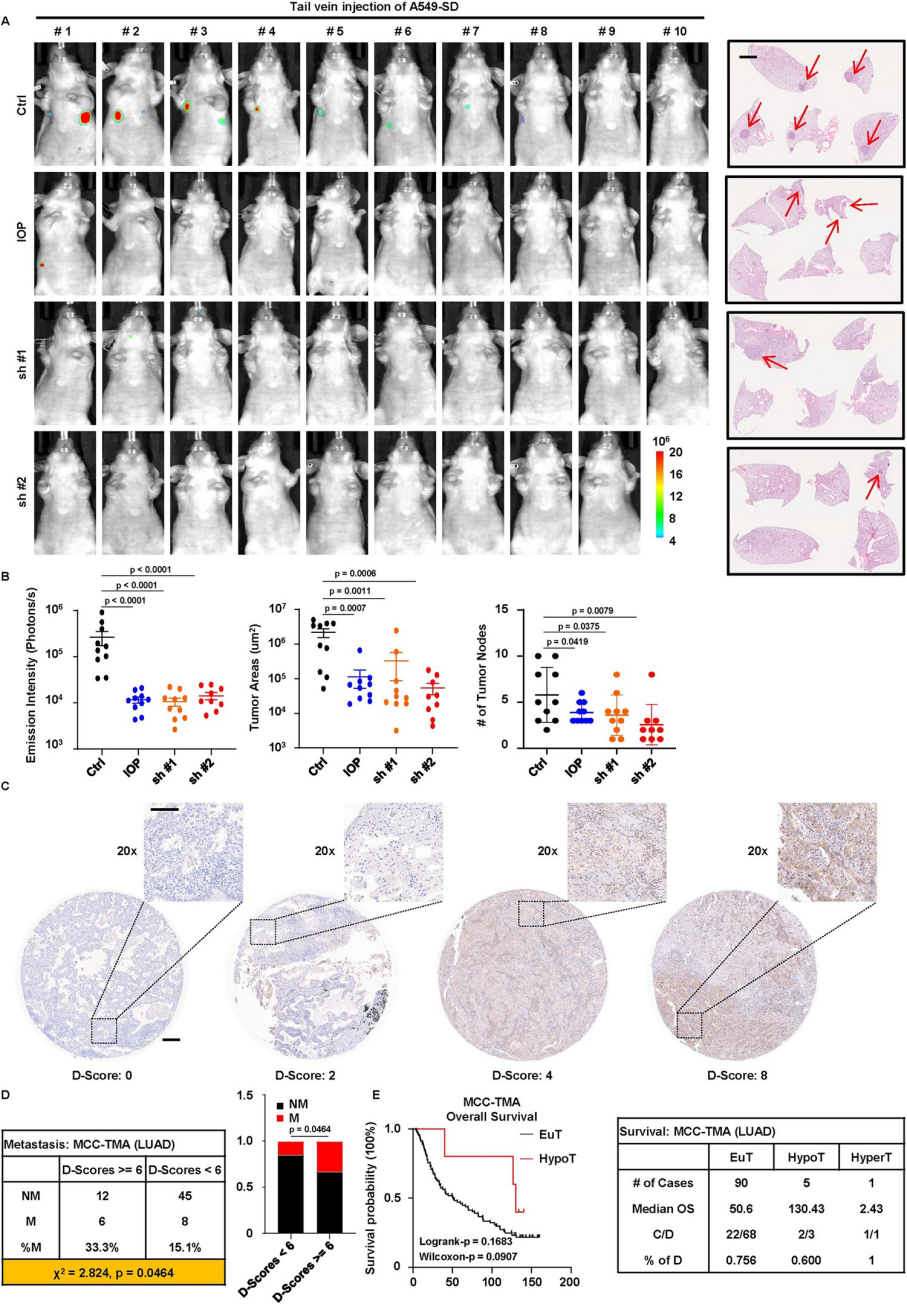
Clinical implication of DIO2-TH signaling. **A,** (Left panel) In vivo imaging of mice intravenously injected with SD cells (Ctrl), and mice injected with SD cells and orally administered with IOP (IOP), and mice injected with SD-shDIO2 cells (sh #1 and sh #2). (Right panel) H&E staining of lung tissue sections of mice. Scale bar, 2mm. Arrows indicate tumor nodules. **B,** Quantification of results in A (n = 10 for Ctrl, IOP, sh #1, and n = 9 for sh #2). Results are shown as mean ± SEM for emission intensity and tumor areas and mean ± SD for tumor nodes. Statistical method: two-tailed Mann-Whitney test (emission intensity and tumor areas); one-tailed unpaired t test (Ctrl-sh #1 and Ctrl-sh #2 in tumor nodes); one-tailed unpaired Welch’s t test (Ctrl-IOP in tumor nodes). **C,** Representative results of IHC) staining of DIO2 on Markey Cancer Center Tissue Microarray (MCC-TMA) of LUAD patients. Scale bar (origin), 2mm. Scale bar (20x), 100μm. **D,** Analysis of metastatic events of MCC-TMA patients. Based on staining scores of DIO2 (D-Scores) suggested by pathologist, patients are separated into two groups: High DIO2 (D-Scores > = 6), Low DIO2 (D-Scores < 6). Metastatic events are characterized by regional or distant metastasis. NM, non-metastatic. M, metastatic. Statistical method: one-sided Chi-square test. **E,** Kaplan-Meier overall survival curve and statistics of euthyroid (EuT) and hypothyroid (HypoT) patients in MCC-TMA. The single hyperthyroid (HyperT) case is listed in the statistics table as well. C, censored. D, deaths. Statistical methods: Log-rank test and Gehan-Breslow-Wilcoxon test.

To validate the metastasis-promoting effect of DIO2 in vivo, we stably depleted DIO2 in SD cells and introduced luciferase expression in them (hereafter referred to as SD-shDIO2). Next, we intravenously injected SD and SD-shDIO2 cells into nude mice to determine whether depletion of DIO2 or IOP treatment could attenuate metastasis. As predicted, mice injected with SD cells and treated with IOP, or mice injected with SD-shDIO2 cells consistently displayed fewer metastatic lesions as shown by the in vivo bioluminescence and staining results of lung tissues (Fig 7A and 7B), suggesting suppression of metastasis by targeting DIO2. To seek more clinical support and provide translational values, we obtained LUAD tissue microarray (MCC-TMA, S6 Table) from our cancer center and performed immunohistochemistry staining of DIO2 (Fig 7C). We found that samples expressing a higher level of DIO2 (H-DIO2, D-Scores ≥ = 6) showed a higher rate of metastatic events than samples expressing less DIO2 (L-DIO2, D-Scores < 6) (Fig 7D), consistent with the previous results from TCGA-LUAD (Fig 5D). Interestingly, the MCC-TMA tissue microarray included 7 patients with hypothyroidism (HypoT) and 1 case with hyperthyroidism (HyperT). Together with the remaining cases (treated as euthyroid, EuT), we compared survival outcomes among these three subcohorts. Although not statistically significant, patients in the HypoT group tended to have better survival outcomes than those in the EuT and HyperT groups (Fig 7E). Due to the scarcity of samples with precise information of thyropathies, this finding was not conclusive, admittedly. Despite this fact, our results provided useful insights into the clinical significance of DIO2 and TH in the metastasis and survival of LUAD patients.

## Discussion

**Fig 8:**
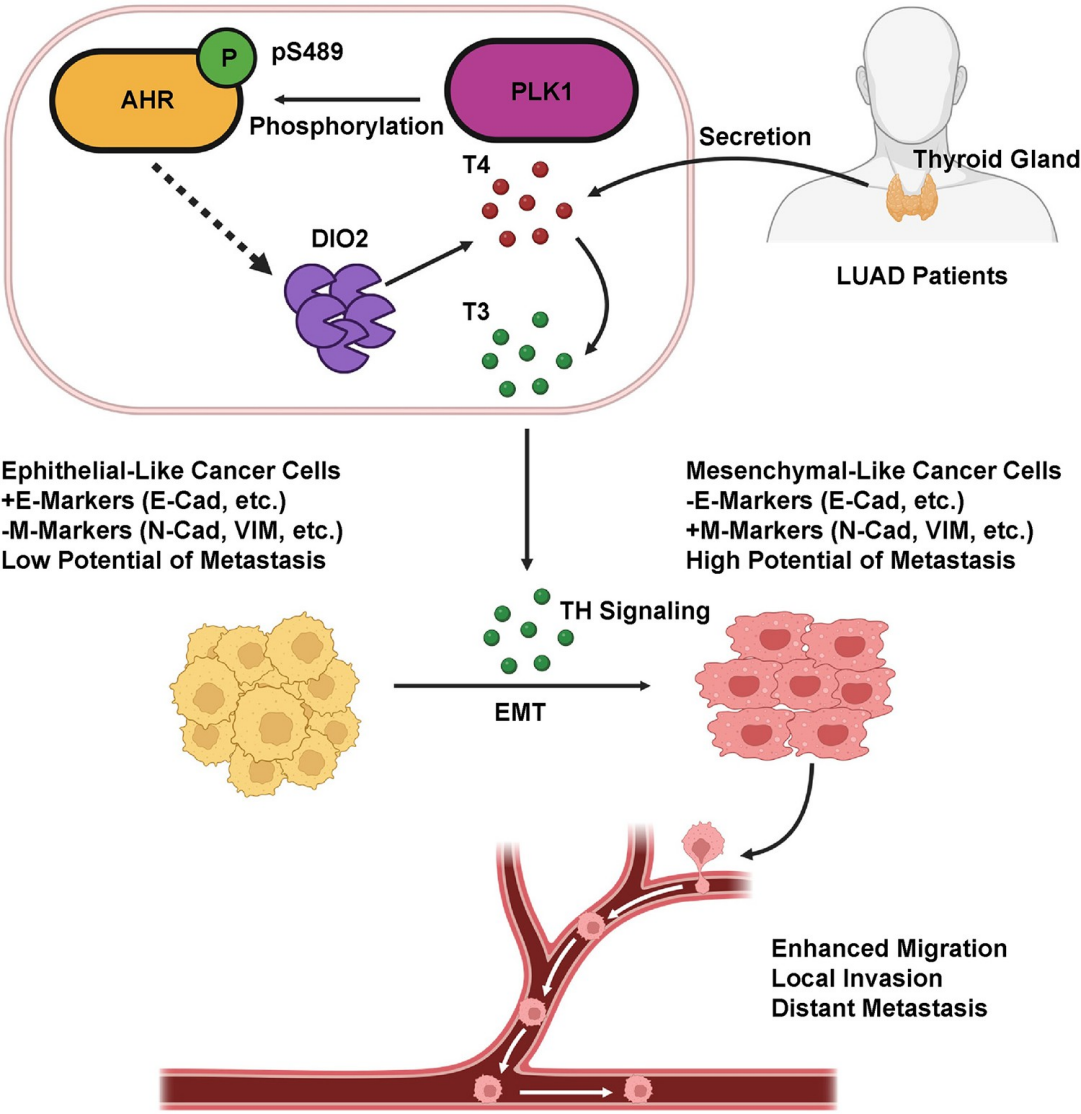
Putative working model. T4 secreted by thyroid glands of LUAD patients enters the circulation system and reaches the local environment of tumors. Phosphorylation of AHR by PLK1 elevates DIO2, which converts the less potent T4 to more potent T3. T3 activates the TH signaling and promotes EMT. Mesenchymal-like tumor cells are prone to local invasion and distant metastasis. Created with BioRender.com.

Specifically for lung cancer, the five-year survival rate for patients with regional diseases is only 30% compared to 60% for patients with localized diseases, and this number further drops to less than 5% for patients with distant metastasis. Sadly, nearly 70% of lung cancer patients have developed metastatic lesions upon diagnosis, underscoring an urgent need for more effective diagnostic techniques to detect the early onset of metastasis, and deeper mechanistic studies to explain this unwanted outcome. In this pursuit, our research offers a compelling hypothesis regarding the metastatic cascade in LUAD (as illustrated in Fig 8). We unveil a critical link wherein PLK1’s phosphorylation of AHR at S489 emerges as a driver of metastasis. Mechanistically, this phosphorylation event triggers the upregulation of DIO2, catalyzing the conversion of T4 to T3, thereby activating the TH signaling pathway, inducing the EMT process, and initiating metastasis. Notably, the identified phosphorylation site resides within the C-terminal region, a locus where significant distinctions between human and mouse AHR have been observed [37]. Coupled with our findings in S1 Fig, we posit that this phosphorylation site may be a unique feature specific to human.

Given PLK1’s well-established role as an oncogene across various cancer types, our sustained interest has been drawn to the potential of targeting PLK1 for cancer therapy. Numerous studies have already illuminated the promise of such an approach, demonstrating its efficacy [18,38–40]. Nevertheless, early clinical trials employing anti-PLK1 monotherapy or combination treatments faced setbacks due to treatment-related toxicity [41]. This challenge spurred the development of more refined PLK1 inhibitors, strategically designed to selectively target cancer cells. Notably, our experiments utilize the PLK1 inhibitor ONV, which is currently undergoing phase 2 clinical trials [42]. While there is a dearth of documented clinical studies about targeting PLK1 in LUAD, our investigation into the interplay between PLK1, AHR phosphorylation, and metastasis in LUAD has yielded vital evidence supporting the rationale for targeting PLK1 in LUAD patients’ treatment. We eagerly anticipate the potential initiation of clinical trials, building upon our preclinical findings and those of our peers, to further validate this therapeutic strategy.

AHR has been shown to account for lung cancer carcinogenesis [43,44]. However, studies regarding its role in lung cancer metastasis have yielded conflicting conclusions [45]. It is noteworthy that AHR’s pro-metastatic function has been substantiated in clinical contexts, where elevated AHR levels correlate positively with disease recurrence and distant metastasis in LUAD patients [23]. This observation aligns seamlessly with our experimental results, where AHR phosphorylation serves to amplify DIO2 expression, thereby promoting metastatic processes. Our research has unveiled intriguing facets of this interaction, suggesting that AHR phosphorylation might modulate its transcriptional activity while concurrently upregulating DIO2. However, the precise mechanisms through which AHR phosphorylation exerts control over its transcriptional activity remain ripe for exploration. While our in silico evidence hints at a potential blockade of ligand-binding capabilities through phosphorylation, the need for further empirical validation is evident. Moreover, it’s plausible that other mechanisms, such as interference with nuclear translocation, or crosstalk with other co-factors and pathways [46,47], may contribute to the dampening of AHR activity upon PLK1-mediated phosphorylation, and this possibility remains to be explored. Another important note is that our investigations have centered on LUAD, and the biological significance of the PLK1-AHR interaction in other lung cancer subtypes remains to be studied. It’s worth emphasizing that a significant portion of lung cancer cases, approximately 40%, comprises LUSC and SCLC [48]. Regrettably, our understanding of the roles of PLK1 and AHR in these subtypes is relatively limited. Therefore, future research needs to dissect the potential molecular functions of PLK1 and AHR in these less explored lung cancer variants.

While DIO2 has emerged as a recent focus in metastasis research [36], its roles in other cancer types have remained underexplored. To our knowledge, our study stands as the pioneering effort to unveil the premetastatic implications of DIO2 in LUAD. As a pivotal enzyme within the TH metabolism, DIO2 plays a central role in converting T4 to the more potent T3. Notably, T3 activation subsequently triggers TH signaling, thereby fostering the EMT process and metastasis. By drawing comparisons among LUAD patients with varying thyropathies, we posit the intriguing notion that hypothyroidism may bear favorable prognostic significance for lung cancer patients. This conjecture aligns with earlier reports indicating improved overall survival among NSCLC patients with hypothyroidism compared to euthyroid counterparts [49]. Nevertheless, we acknowledge the preliminary nature of this hypothesis, as our access to patients with thyroid complications has been limited, constraining our ability to draw definitive conclusions. Beyond DIO2’s well-established role in metastasis promotion, we also note its potential involvement in other fundamental cancer hallmarks, such as immune evasion within the context of lung cancer [50]. Alongside its intricate associations with AHR and PLK1, a comprehensive exploration of these multifaceted aspects promises to enhance our comprehension of lung cancer etiology, ultimately translating into improved clinical care for patients.

## Supporting information

S1 Fig Mouse Ahr is not phosphorylated by mouse Plk1 at consensus site. **A,** Alignment of consensus sequences around AHR S489 in different species. **B,** IB to detect p-Ahr in KP and KPP cells, which are two mouse lung adenocarcinoma cell lines.
(TIF)

S2 Fig AHR and PLK1 are important for LUAD progression. **A,** Comparison of AHR and PLK1 expressions among normal lung, LUAD, LUSC, and SCLC cell lines using data from the DepMap database. Statistical methods: Welch’s ANOVA test following multiple comparisons (AHR); one-way ANOVA following multiple comparisons (PLK1). **B,** Kaplan-Meier overall survival curve of 79 SCLC patients from shared dataset (George et al.). Patients are separated into four groups: H-PLK1/H-AHR (PLK1/AHR > median), H-PLK1/L-AHR (PLK1 > median/AHR < median), L-PLK1/H-AHR (PLK1 < median/AHR > median), L-PLK1/L-AHR (PLK1/AHR < median). Statistical method: Log-rank test. **C,** Kaplan-Meier survival curves of TCGA-LUSC patients separated into four groups: H-PLK1/H-AHR (PLK1/AHR > median), H-PLK1/L-AHR (PLK1 > median/AHR < median), L-PLK1/H-AHR (PLK1 < median/AHR > median), L-PLK1/L-AHR (PLK1/AHR < median). Statistical method: Log-rank test. **D,** Comparison of AHR and PLK1 expressions (RSEM) between TCGA-LUAD and TCGA-LUSC patients. Statistical method: two-tailed unpaired Welch’s t test. **E,** Kaplan-Meier survival curves of TCGA-LUAD and TCGA-LUSC patients after adjusting thresholds. In both datasets, thresholds are set as the median expression (RSEM) of PLK1 in TCGA-LUSC and median expression (RSEM) of AHR in TCGA-LUAD. Adjusted thresholds different from previous results are marked with asterisks (H-PLK1* and L-PLK1* in TCGA-LUAD, H-AHR* and L-AHR* in TCGA-LUSC). Statistical method: Log-rank test.
(TIF)

S3 Fig PLK1 phosphorylates AHR to promote metastasis of LUAD. **A,** Wound healing assays with TET-PLK1 and TET-shPLK1 A549 cells. D+O, DOX plus ONV. The final concentrations used are 200ng/ml for DOX and 50nM for ONV. Results are normalized to 0h and shown as mean ± SD (n = 3). Scale bar, 250μm. Statistical method: two-tailed unpaired t test. **B,** 3D spheroid formation and 3D growth assays with V, WT, SA, SD cells. Results are normalized to V and shown as mean ± SD (n = 11 for spheroid formation and n = 6 for 3D growth). Scale bar, 20μm. Statistical method: one-way ANOVA test. **C,** Gating strategy of cell cycle analysis by flow cytometry. 10^4^ cells are counted for each group.
(TIF)

S4 Fig Metastasis assays with H1299 cells. A, IB to verify establishment of H1299 V, WT, SA, SD cells. B, C, Wound healing assay with H1299 V, WT, SA, SD cells. Results are normalized to 0h. Scale bar, 100μm. Statistical method: two-tailed unpaired t test. D, 3-day 2D growth assay with H1299 V, WT, SA, SD cells. Results are normalized to day 0 and shown as mean ± SD (n = 8). Statistical method: one-way ANOVA test following multiple comparisons. E, Transwell migration, invasion, 3D invasion and spheroid formation assays with H1299 V, WT, SA SD cells. Results are normalized to V and shown as mean ± SD (n = 3 for transwell migration and invasion assays, n = 12 for 3D invasion assay, n = 11 for spheroid formation). Scale bar (upper two panels), 100μm. Scale bar (bottom two panel), 20μm. Statistical methods: two-tailed unpaired t test (transwell); two-tailed Mann-Whitney test (3D invasion); Kruskal-Wallis test following multiple comparisons (3D sphere). F, 3D growth assay with H1299 V, WT, SA, SD cells. Results are normalized to V and shown as mean ± SD (n = 6). Statistical method: Kruskal-Wallis test following multiple comparisons. G, Cell cycle analysis of H1299 V, WT, SA, SD cells by flow cytometry. Results are normalized to V and shown as mean ± SD (n = 3).
(TIF)

S5 Fig Gating strategy of flow cytometry analysis of circulating GFP^+^ tumor cells. Each sample is enriched with 10^3^ GFP^+^ cells and the percentage of GFP^+^ tumor cells is calculated.
(TIF)

S6 Fig RNA-seq analysis and validation. **A,** Dotplot (SD vs SA) of significantly upregulated pathways in SD cells (q values < 0.05), identified by GSEA with hallmark gene set. No significantly downregulated pathways were identified. **B,** Enrichment plots of EMT pathway. **C,** Spearman correlation analysis (Log_2_TPM) of DIO2 and PLK1 in normal lung samples and tumor samples from TCGA-LUAD. **D,** Expressions (Log_2_TPM) of DIO2 and PLK1 between normal lung samples and tumor samples, as well as among different stages of tumor samples, from TCGA-LUAD. Statistical methods: linear-mixed model test (left panel); one-tailed Mann-Whitney test (right panel). **E, F,** Kaplan-Meier survival curves of TCGA-LUAD patients. Patients are separated into two groups: H-DIO2 (DIO2 > = 3^rd^ quartile) and L-DIO2 (DIO2 < = 1^st^ quartile). Statistical method: Log-rank test.
(TIF)

S7 Fig Spearman correlation analysis of DIO2 and EMT markers in TCGA-LUAD. A, Correlation analysis (Log2TPM) of DIO2 and M-Markers. B, Correlation analysis (Log2TPM) of DIO2 and E-Markers.
(TIF)

S8 Fig AHR phosphorylation is associated with its activity and DIO2 expression. **A,** 3D animated images of WT AHR and phospho-S489 AHR. **B,** 3D superimposed image of PAS-B domains (275–386) from WT AHR and phospho-S489 AHR. **C,** Radius of Gyration (RoG) simulation of PAS-B domains from WT AHR and phospho-S489 AHR. **D,** Results of Solvent-Accessible Surface Area (SASA) simulation of PAS-B domains from WT AHR and phospho-S489 AHR. **E,** qPCR to detect the expressions of AHR downstream targets in WT, SA, and SD cells. Results of qPCR are normalized to WT and shown as mean ± SD (n = 3). Statistical methods: one-tailed unpaired Welch’s t test (SD-SA for CYP1A1, WT-SA for CYP1A2); one-tailed unpaired t test (rest comparisons). **F,** IB to detect DIO2 in A549 cells treated with 20μM AHR inhibitor CH-223191 (CH) for 48 hours.
(TIF)

S9 Fig DIO2 promotes metastasis of LUAD via TH signaling. **A,** Wound healing assay with A549 cells treated with T3, T4, IOP, T3+I, T4+I. The final concentrations used are 10nM for T3 and T4, and 50μM for IOP. Results are normalized to 0h and shown as mean ± SD (n = 3). Scale bar, 250μm. Statistical method: two-tailed unpaired t test. **B,** 3-day 2D cell growth assay with A549 cells treated with T3, T4, IOP, T3+I, T4+I. The final concentrations used are 10nM for T3 and T4, and 50μM for IOP. Results are normalized to day 0 and shown as mean ± SD (n = 8). Statistical method: Kruskal-Wallis test. **C,** Wound healing assay with SD cells treated with 50μM IOP or transiently depleting DIO2 by siRNA (si #1, si #2). Results are normalized to 0h and shown as mean ± SD (n = 3). Scale bar, 250μm. Statistical method: two-tailed unpaired t-test. **D,** IB to detect EMT markers in SD cells transiently depleting DIO2 by siRNA. **E,** Transwell migration and invasion assays with SD cells treated with 50μM IOP or depleting DIO2 by siRNA. Results are normalized to Ctrl and shown as mean ± SD (n = 3 for both assays). Scale bar, 100μm. Statistical method: two-tailed unpaired t test. **F,** Wound healing assay with SD cells depleting DIO2 by shRNA. Results are normalized to 0h and shown as mean ± SD (n = 3). Scale bar, 250μm. Statistical method: two-tailed unpaired t test. **G,** 3-day 2D cell growth assay with A549 cells treated with 50μM IOP or depleting DIO2 by shRNA. Results are normalized to day 0 and shown as mean ± SD (n = 8). Statistical method: Welch’s ANOVA test following multiple comparisons.
(TIF)

S1 Table Survival summary of LUAD and LUSC patients before adjusting thresholds. (XLSX)

S2 Table Survival summary of LUAD and LUSC patients after adjusting thresholds. (XLSX)

S3 Table GSEA results of RNA-seq. (XLSX)

S4 Table Gene list with expression order of SD > WT > SA ≈ V and their correlation with PLK1 in TCGA-LUAD. (XLSX)

S5 Table Survival summary of H-DIO2 and L-DIO2 patients in TCGA-LUAD. (XLSX)

S6 Table Information of MCC-TMA patients (metastasis and survival). (XLSX)

S7 Table Numerical data for rebuilding figures. (XLSX)

S1 Appendix Additional methods and reagent information. (PDF)

S2 Appendix Immunoblot replicates. (PDF)
