# Supplementary figures and images for: Phosphorylation of AHR by PLK1 promotes metastasis of LUAD via DIO2-TH signaling
Source: PLoS Genet. 2023 Nov 21;19(11):e1011017. doi: 10.1371/journal.pgen.1011017 (PMC10662729; doi:10.1371/journal.pgen.1011017)

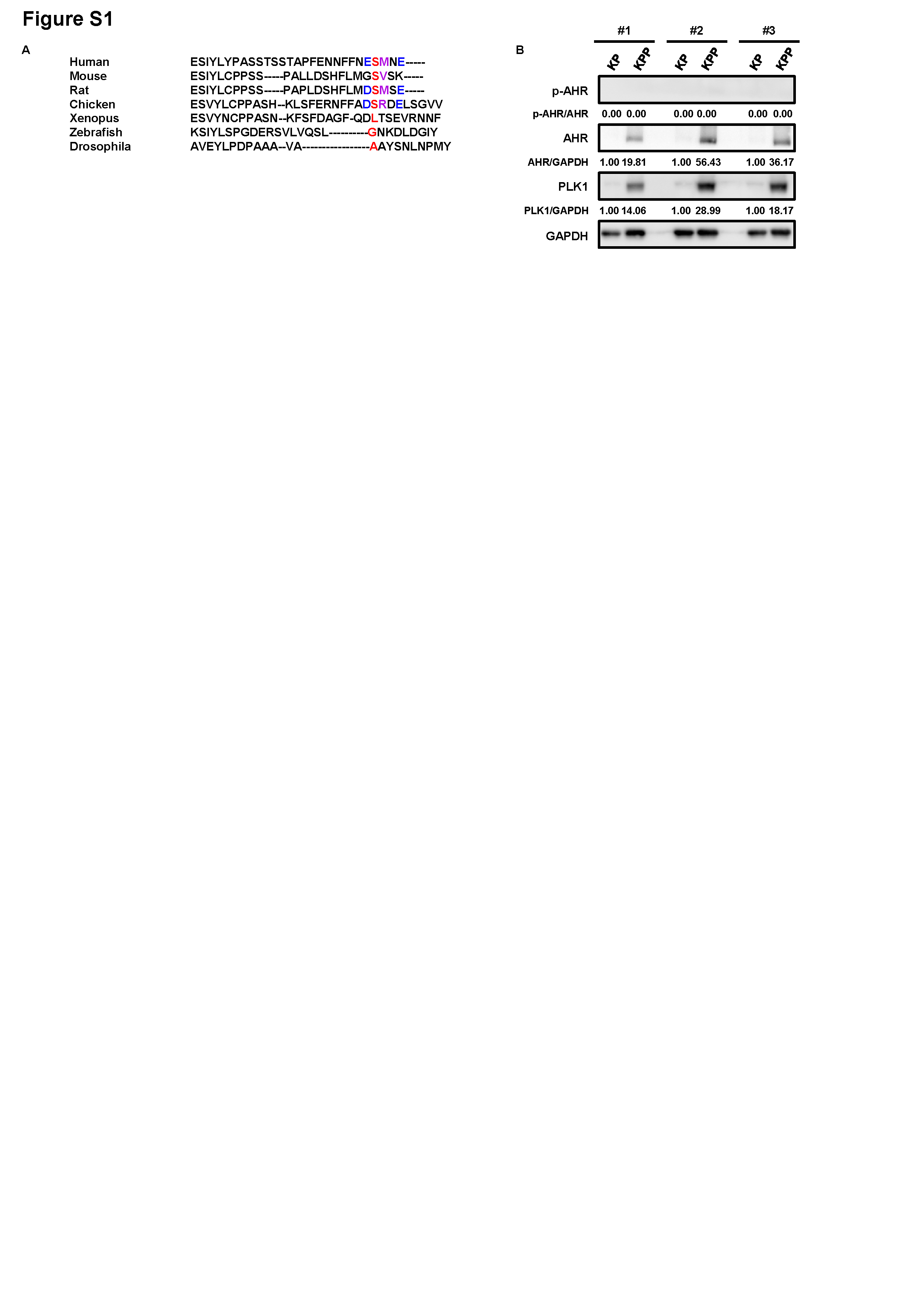

Supplement: S1 Fig — A, Alignment of consensus sequences around AHR S489 in different species. B, IB to detect p-Ahr in KP and KPP cells, which are two mouse lung adenocarcinoma cell lines. (TIF) [file pgen.1011017.s001.tif]

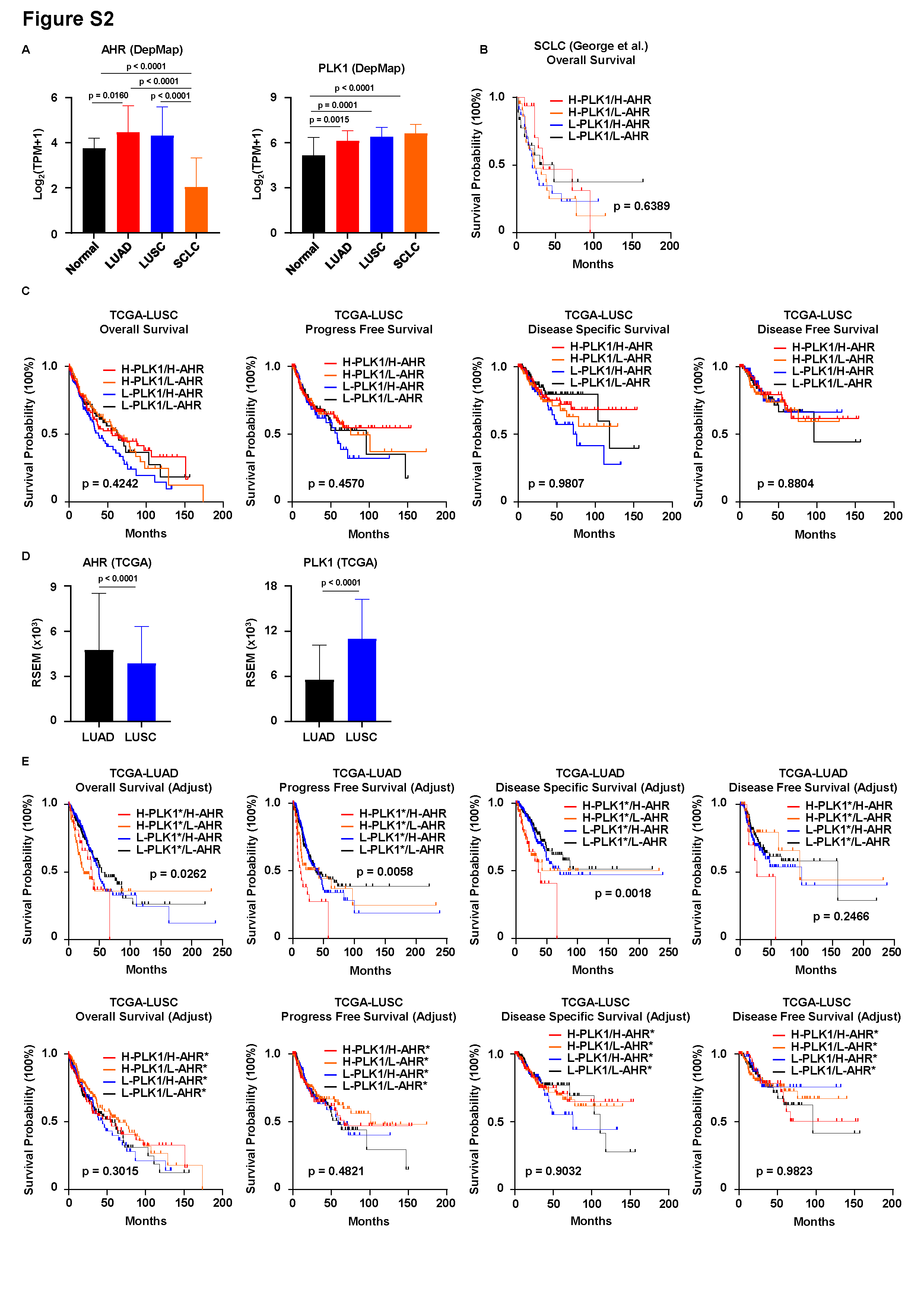

Supplement: S2 Fig — A, Comparison of AHR and PLK1 expressions among normal lung, LUAD, LUSC, and SCLC cell lines using data from the DepMap database. Statistical methods: Welch’s ANOVA test following multiple comparisons (AHR); one-way ANOVA following multiple comparisons (PLK1). B, Kaplan-Meier overall survival curve of 79 SCLC patients from shared dataset (George et al.). Patients are separated into four groups: H-PLK1/H-AHR (PLK1/AHR > median), H-PLK1/L-AHR (PLK1 > median/AHR < median), L-PLK1/H-AHR (PLK1 < median/AHR > median), L-PLK1/L-AHR (PLK1/AHR < median). Statistical method: Log-rank test. C, Kaplan-Meier survival curves of TCGA-LUSC patients separated into four groups: H-PLK1/H-AHR (PLK1/AHR > median), H-PLK1/L-AHR (PLK1 > median/AHR < median), L-PLK1/H-AHR (PLK1 < median/AHR > median), L-PLK1/L-AHR (PLK1/AHR < median). Statistical method: Log-rank test. D, Comparison of AHR and PLK1 expressions (RSEM) between TCGA-LUAD and TCGA-LUSC patients. Statistical method: two-tailed unpaired Welch’s t test. E, Kaplan-Meier survival curves of TCGA-LUAD and TCGA-LUSC patients after adjusting thresholds. In both datasets, thresholds are set as the median expression (RSEM) of PLK1 in TCGA-LUSC and median expression (RSEM) of AHR in TCGA-LUAD. Adjusted thresholds different from previous results are marked with asterisks (H-PLK1* and L-PLK1* in TCGA-LUAD, H-AHR* and L-AHR* in TCGA-LUSC). Statistical method: Log-rank test. (TIF) [file pgen.1011017.s002.tif]

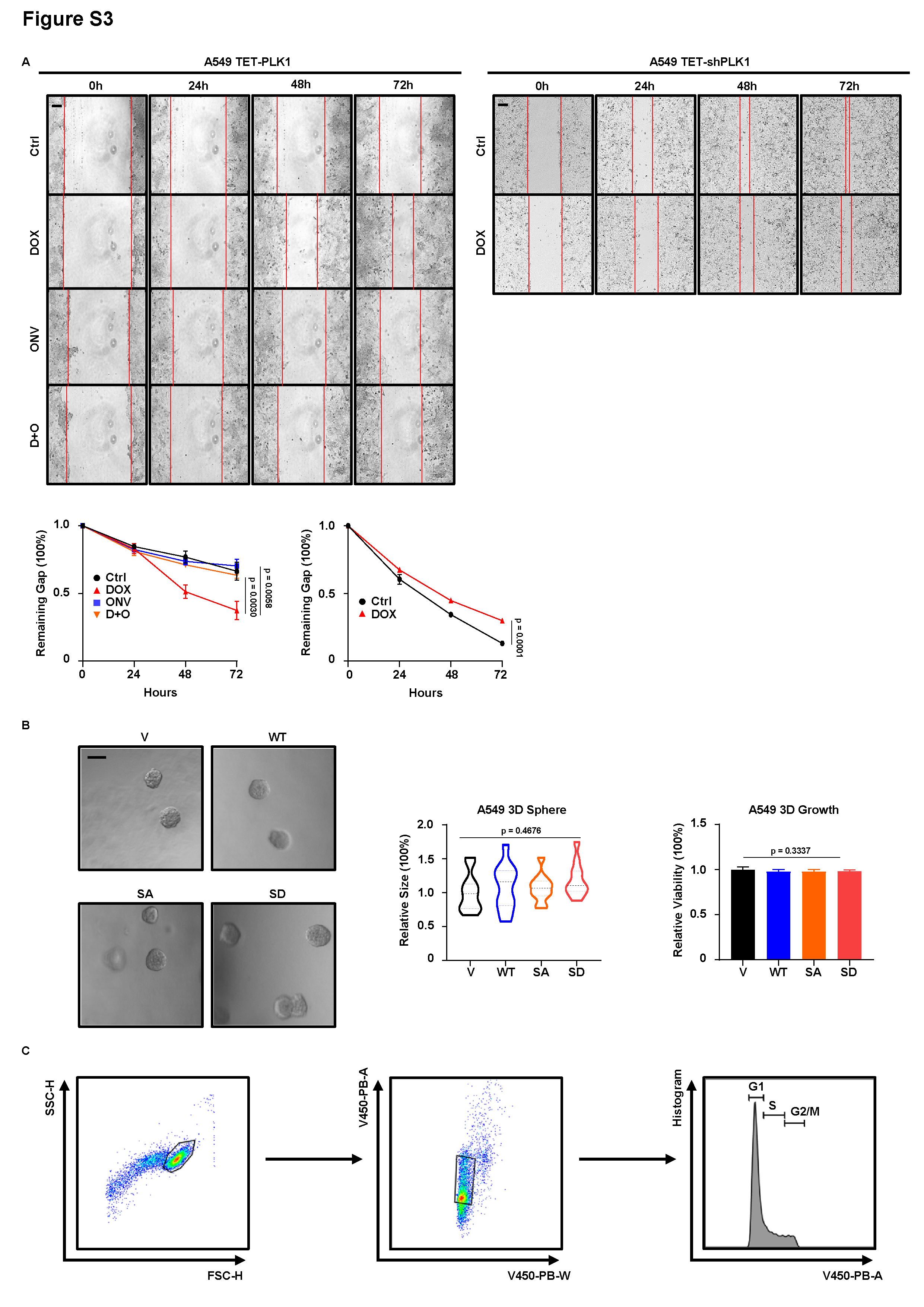

Supplement: S3 Fig — A, Wound healing assays with TET-PLK1 and TET-shPLK1 A549 cells. D+O, DOX plus ONV. The final concentrations used are 200ng/ml for DOX and 50nM for ONV. Results are normalized to 0h and shown as mean ± SD (n = 3). Scale bar, 250μm. Statistical method: two-tailed unpaired t test. B, 3D spheroid formation and 3D growth assays with V, WT, SA, SD cells. Results are normalized to V and shown as mean ± SD (n = 11 for spheroid formation and n = 6 for 3D growth). Scale bar, 20μm. Statistical method: one-way ANOVA test. C, Gating strategy of cell cycle analysis by flow cytometry. 104 cells are counted for each group. (TIF) [file pgen.1011017.s003.tif]

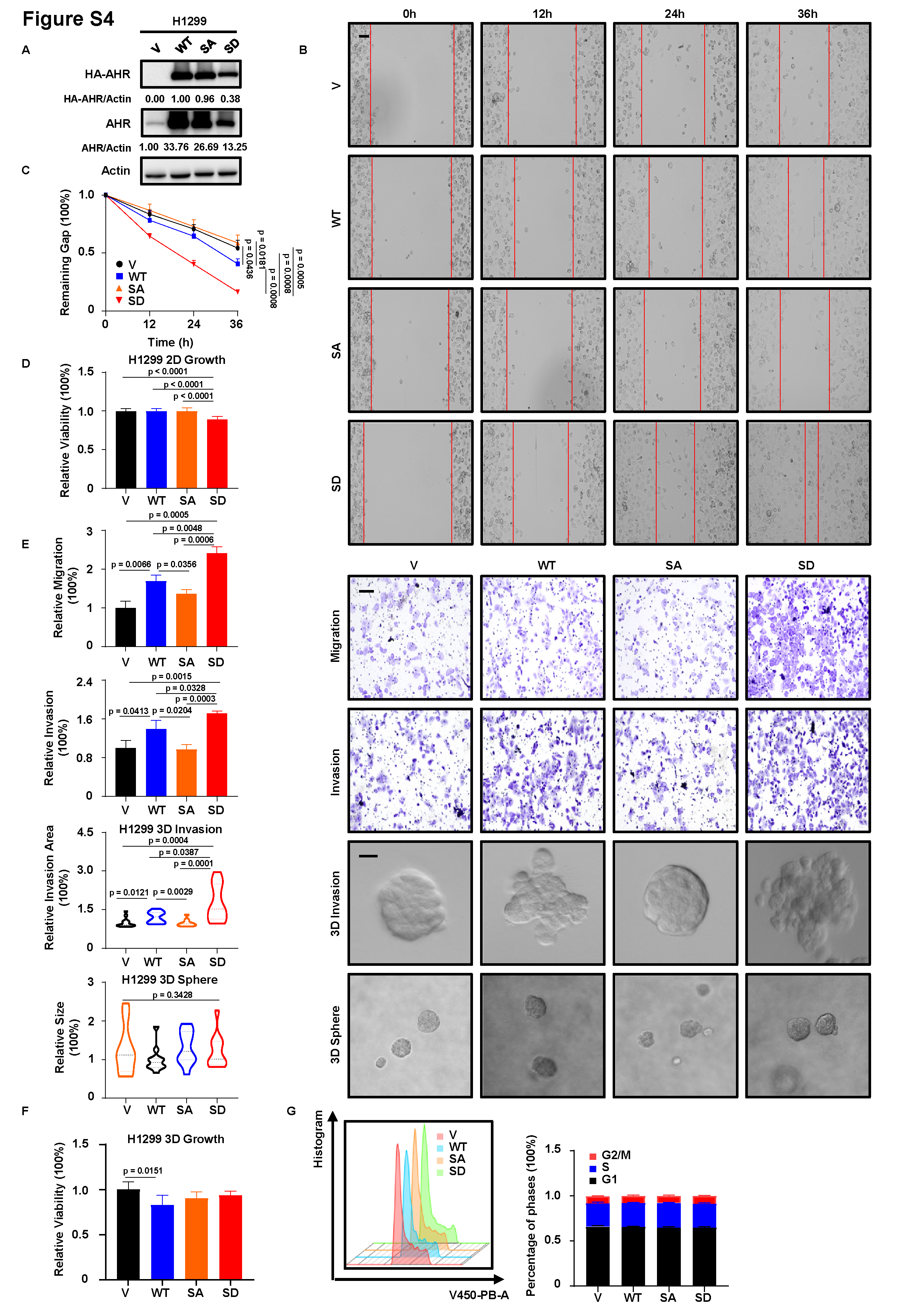

Supplement: S4 Fig — A, IB to verify establishment of H1299 V, WT, SA, SD cells. B, C, Wound healing assay with H1299 V, WT, SA, SD cells. Results are normalized to 0h. Scale bar, 100μm. Statistical method: two-tailed unpaired t test. D, 3-day 2D growth assay with H1299 V, WT, SA, SD cells. Results are normalized to day 0 and shown as mean ± SD (n = 8). Statistical method: one-way ANOVA test following multiple comparisons. E, Transwell migration, invasion, 3D invasion and spheroid formation assays with H1299 V, WT, SA SD cells. Results are normalized to V and shown as mean ± SD (n = 3 for transwell migration and invasion assays, n = 12 for 3D invasion assay, n = 11 for spheroid formation). Scale bar (upper two panels), 100μm. Scale bar (bottom two panel), 20μm. Statistical methods: two-tailed unpaired t test (transwell); two-tailed Mann-Whitney test (3D invasion); Kruskal-Wallis test following multiple comparisons (3D sphere). F, 3D growth assay with H1299 V, WT, SA, SD cells. Results are normalized to V and shown as mean ± SD (n = 6). Statistical method: Kruskal-Wallis test following multiple comparisons. G, Cell cycle analysis of H1299 V, WT, SA, SD cells by flow cytometry. Results are normalized to V and shown as mean ± SD (n = 3). (TIF) [file pgen.1011017.s004.tif]

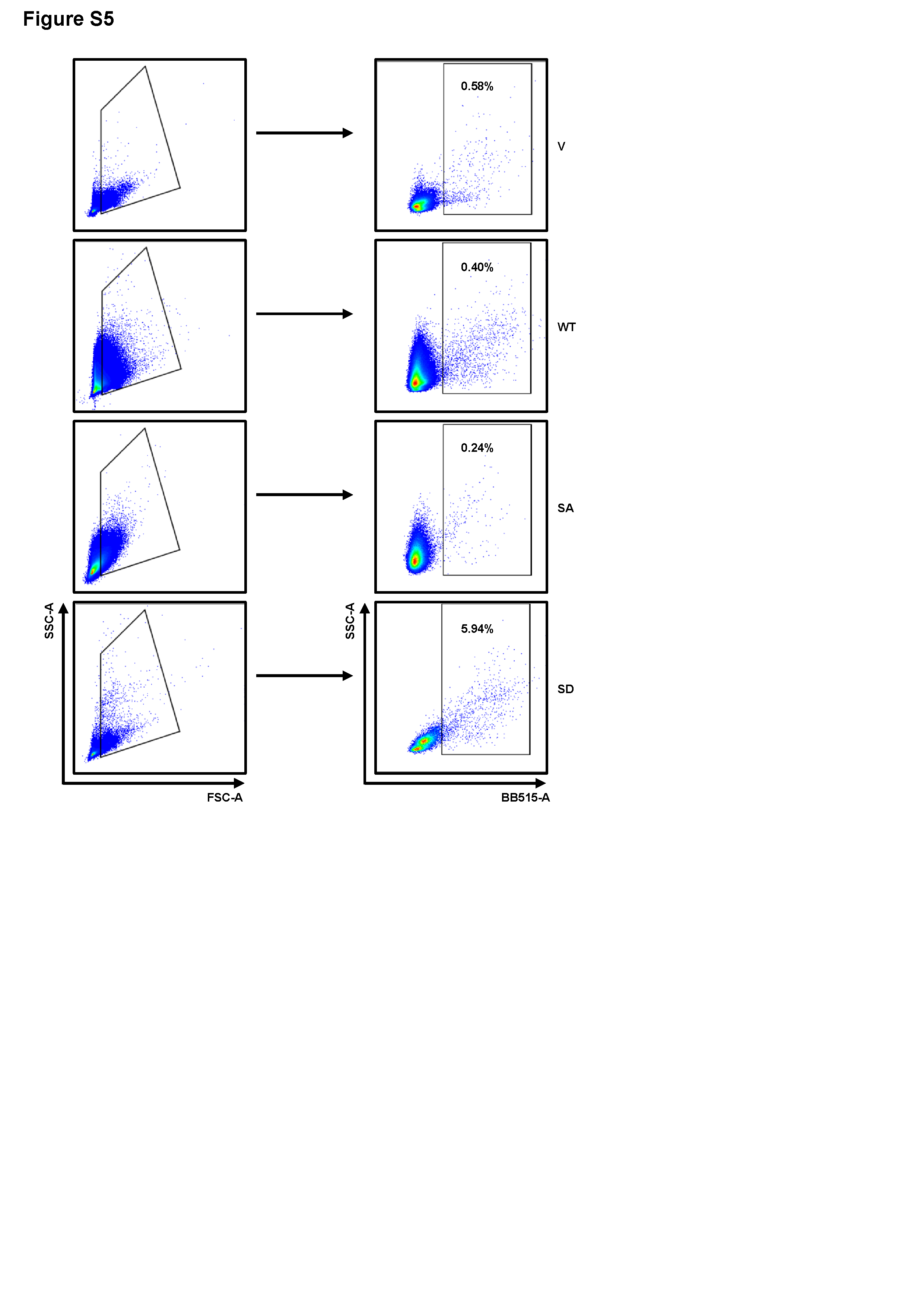

Supplement: S5 Fig — Each sample is enriched with 103 GFP+ cells and the percentage of GFP+ tumor cells is calculated. (TIF) [file pgen.1011017.s005.tif]

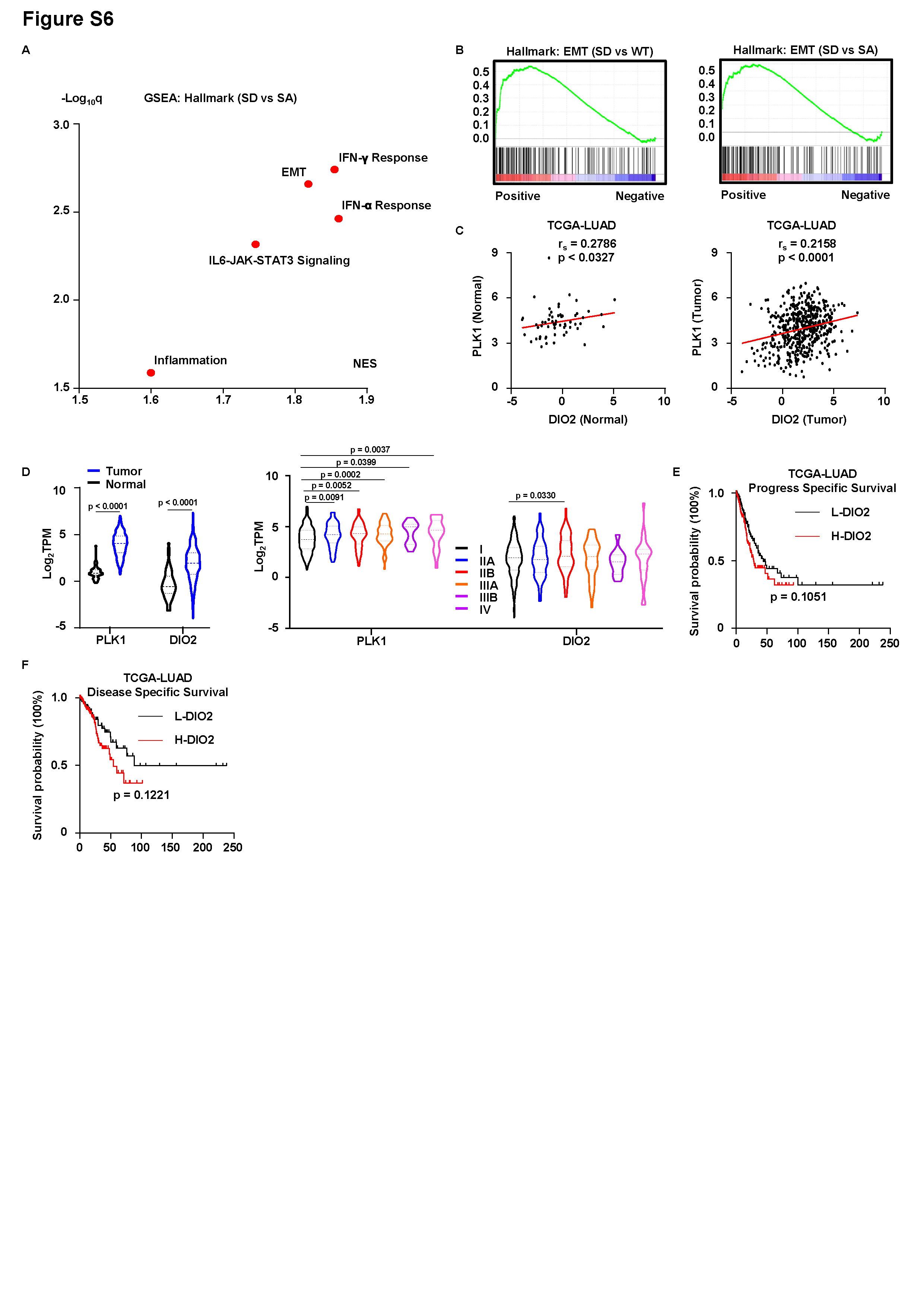

Supplement: S6 Fig — A, Dotplot (SD vs SA) of significantly upregulated pathways in SD cells (q values < 0.05), identified by GSEA with hallmark gene set. No significantly downregulated pathways were identified. B, Enrichment plots of EMT pathway. C, Spearman correlation analysis (Log2TPM) of DIO2 and PLK1 in normal lung samples and tumor samples from TCGA-LUAD. D, Expressions (Log2TPM) of DIO2 and PLK1 between normal lung samples and tumor samples, as well as among different stages of tumor samples, from TCGA-LUAD. Statistical methods: linear-mixed model test (left panel); one-tailed Mann-Whitney test (right panel). E, F, Kaplan-Meier survival curves of TCGA-LUAD patients. Patients are separated into two groups: H-DIO2 (DIO2 > = 3rd quartile) and L-DIO2 (DIO2 < = 1st quartile). Statistical method: Log-rank test. (TIF) [file pgen.1011017.s006.tif]

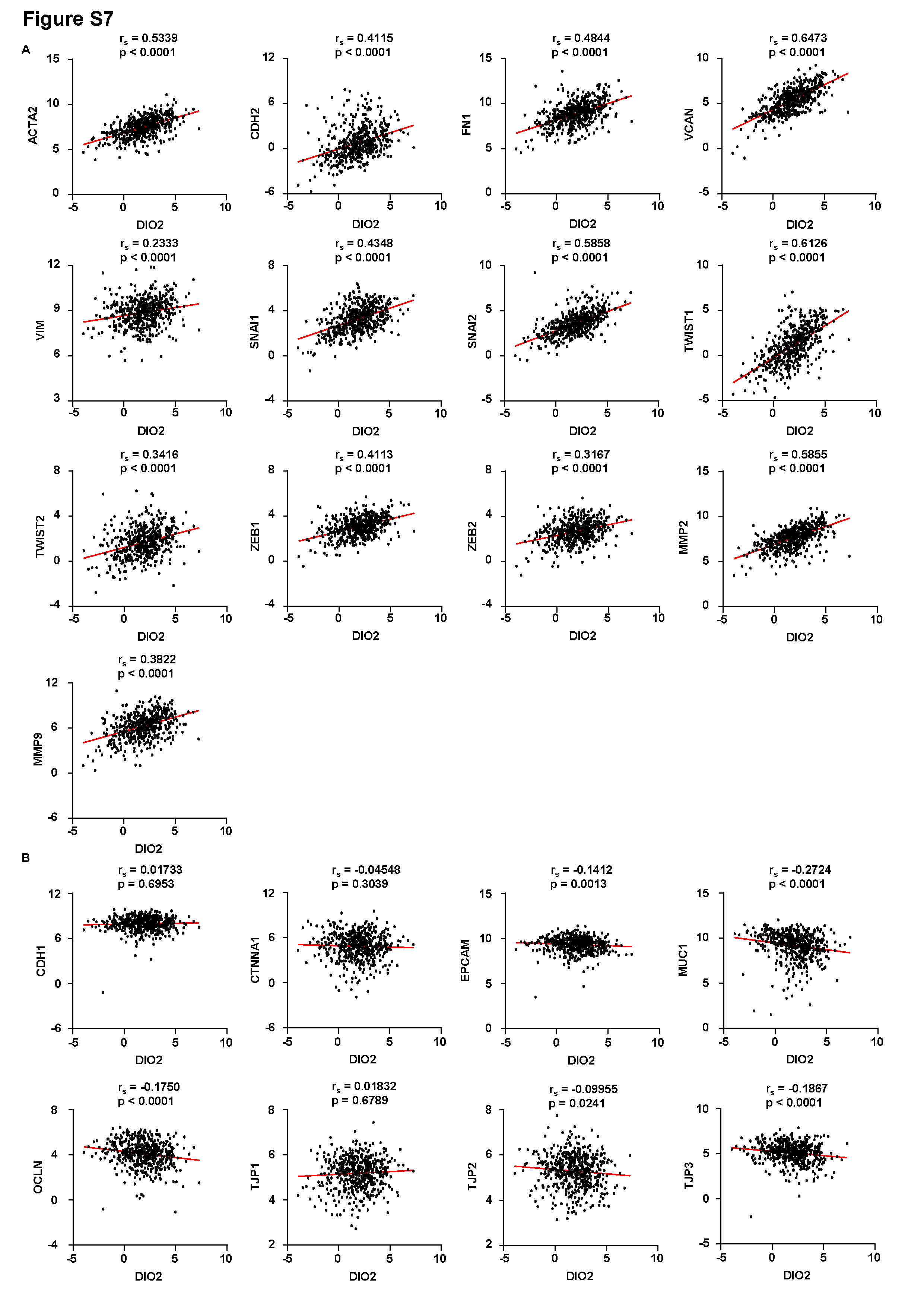

Supplement: S7 Fig — A, Correlation analysis (Log2TPM) of DIO2 and M-Markers. B, Correlation analysis (Log2TPM) of DIO2 and E-Markers. (TIF) [file pgen.1011017.s007.tif]

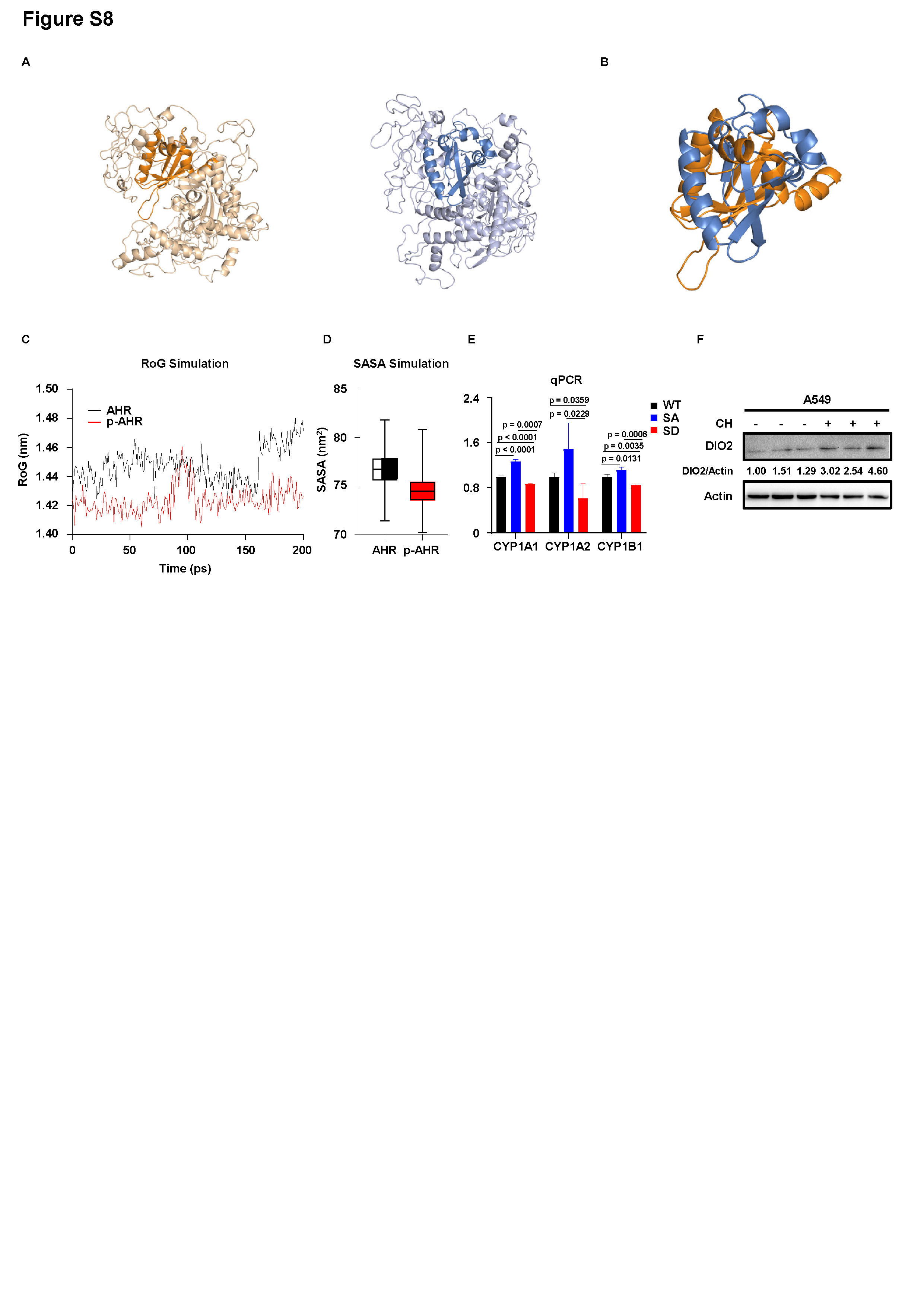

Supplement: S8 Fig — A, 3D animated images of WT AHR and phospho-S489 AHR. B, 3D superimposed image of PAS-B domains (275–386) from WT AHR and phospho-S489 AHR. C, Radius of Gyration (RoG) simulation of PAS-B domains from WT AHR and phospho-S489 AHR. D, Results of Solvent-Accessible Surface Area (SASA) simulation of PAS-B domains from WT AHR and phospho-S489 AHR. E, qPCR to detect the expressions of AHR downstream targets in WT, SA, and SD cells. Results of qPCR are normalized to WT and shown as mean ± SD (n = 3). Statistical methods: one-tailed unpaired Welch’s t test (SD-SA for CYP1A1, WT-SA for CYP1A2); one-tailed unpaired t test (rest comparisons). F, IB to detect DIO2 in A549 cells treated with 20μM AHR inhibitor CH-223191 (CH) for 48 hours. (TIF) [file pgen.1011017.s008.tif]
