# Supplementary material for: Phosphorylation of AHR by PLK1 promotes metastasis of LUAD via DIO2-TH signaling
Source: PLoS Genet. 2023 Nov 21;19(11):e1011017. doi: 10.1371/journal.pgen.1011017 (PMC10662729; doi:10.1371/journal.pgen.1011017)
Supplement: S1 Appendix — (PDF) [file pgen.1011017.s017.pdf]

Supplementary materials and methods

Chaohao Li, Daheng He, Ibrahim A. Imam, Qing Shao, Chi Wang and Xiaoqi Liu

Analysis of RNA-seq

Sequencing reads were trimmed and filtered using Trimmomatic (v0.39). Trimmed reads were mapped to the human reference genome assembly GRCh38 transcripts annotation using RSEM [1]. RSEM results normalization and differential expression analysis were performed using the R package edgeR [2]. Transcript per million (TPM) were calculated for each gene [3]. Gene set enrichment analyses (GSEA) were performed on default values calculated with edgeR, between SD vs WT and SD vs SA groups. MSigDB (v7.5) hallmark gene set and GSEA software (v.4.3.2) were used in the analyses with 1000 permutations [4-6], and no collapse was applied. The significantly upregulated and downregulated pathways (q values < 0.05) from GSEA analysis were shown in dotplot. Further filtering genes with the condition of SD > WT > SA = V was performed as follows: Genes with $\log_2$(foldchange) > $\log_2$(1.0) were identified as overexpressed in the comparison of SD vs WT (with WT as reference). Similar filtering was repeated for the comparison of WT vs SA (with SA as reference). The overlapping over-expressed genes in the two filtering were identified as satisfying the condition SD > WT > SA simultaneously. In addition, in the comparison of SA vs V, the genes with raw p values greater than or equal to 0.05 were identified as having little difference between the two groups of comparison. The final list was ranked with q values in the comparison of SD vs WT. For correlation, survival, metastasis and expression analyses, the datasets were downloaded from cBioportal [7-9].

Simulation of AHR and p-AHR

The initial protein configuration of the wild-type AHR was gained from the AlphaFold Protein Structure Database [10], and the initial protein configuration of phospho-S489 AHR at S489 was gained from the Vienna-PTM 2.0 web server [11]. The simulation systems were created by placing a wild-type AHR or p-AHR protein in the center of a 24-nm cubic model box and surrounded by a 3.0-nm water shell. Na^+ ions were used as the counter-ions to make the system neutral in charge. The GROMOS 54A8 force field was used to describe the bonded (stretching, angle, dihedral angle) and non-bonded (Van der Waals force, electrostatic force) interactions[12]. GROMACS (ver. 2022.1) was used to perform energy minimization and molecular dynamics (MD) simulations for all systems [13]. Each simulation system underwent energy minimization to eliminate close contact between atoms. A 200-ns canonical ensemble molecular MD was performed at 310K to achieve thermodynamic equilibrium and record the trajectory after every 200 ps for another 200 ns. A 1.2nm cutoff was implemented for short-range van der Waals forces, and long-range electrostatic forces were calculated using the Particle Mesh Ewald method [14]. The Berendsen method was utilized to rapidly control temperature and pressure during equilibration [15], while the velocity-rescaling method was employed for temperature control during the MD production step [16].

Plasmids, siRNA, transfection and infection

pGEX-KG vector was purchased from ATCC (77103). FLAG-PLK1 and HA-AHR plasmids were previously described [17, 18]. TET-FLAG-PLK1 plasmid was constructed by cloning the coding sequence of human PLK1 onto pCW57-RFP-P2A-MCS vector (Addgene, 78933), which was a gift from Adam Karpf [19]. TET-shPLK1 plasmid was constructed by cloning the PLK1-targeting sequence, which was previously described [17], onto Tet-pLKO-puro vectpr (Addgene, 21915),

which was a gift from Dmitri Wiederschain [20]. pCDH-EF1-Luc2-cG-BSD plasmid (Addgene, 24982) was a gift from Kazuhiro Oka. shRNA plasmids and empty vector were purchased from Sigma. For siRNA-based gene depletion, cells were infected with the siRNA negative control or predesigned siRNA targeting indicated genes, both purchased from Sigma. 48 hours after transfection, cells were harvested for experiments. All transfections were performed using jetPRIME Versatile DNA/siRNA transfection reagent (Polyplus-transfection, 114-15) according to the manufacturer's instructions. For establishment of stable cell lines, lentiviral particles were packaged in 293T cells with psPAX2 (Addgene, 12260) and pCMV-VSV-G (Addgene, 8454), which was a gift from Didier Trono and Bob Weinberg [21], respectively. Infections were performed for 24 hours and cells were subject to resistance selection.

Immunoblotting, immunoprecipitation and immunohistochemistry

Immunoblotting. Cell pellets were washed with ice-cold PBS and 1x RIPA buffers containing 50x protease inhibitor cocktail and 100x phosphatase inhibitor were used for lysis. Pierce BCA Protein Assay Kit (Thermo, 23225) was used to measure concentration of lysates and 15μg total proteins were loaded onto SDS-PAGE gels for electrophoresis. After gel transfer to PVDF membranes, 5% skim milk was used to block the membranes. Primary antibodies in 1x TBST buffer were incubated overnight. After washing three times with 1x TBST buffer, HRP-linked secondary antibodies were applied in 1x TBST buffer for an hour, followed by another three washing with 1x TBST buffer. Bands were probed with SuperSignal West Dura Extended Duration Substrate (Thermo, 34076) and visualized with ChemiDoc Imaging System (Bio-Rad). All experiments were repeated three times (except for Figs. 1F right, 2B) and one representative was shown. Immunoblots were analyzed and quantified by the Image Lab software (Bio-Rad). Results were normalized to one lane. **Immunoprecipitation.** Lysates containing 1mg total protein were

incubated with anti-AHR, anti-PLK1 or IgG control antibodies overnight. The lysates were then incubated with PureProteome Protein A/G Mix Magnetic Beads (Sigma, LSKMAGAG10) at room temperature for 30 mins. After washing the beads with 1x TBST three times, samples were eluted by adding 60ul 1x SDS loading buffer and boiling at 90 °C for 10 mins. The eluates were subject to regular immunoblotting. **Immunohistochemistry.** MCC-TMA slides were acquired from the University of Kentucky Markey Cancer Center. The staining of DIO2 was performed by Biospecimen Procurement And Translational Pathology Shared Resource Facility of Markey Cancer Center. Quality check and scoring of DIO2 was performed by pathologist Dr. Derek Allison.

RNA extraction and qPCR

Total RNA was extracted using RNeasy Mini Kit (Qiagen, 74104) according to manufacturer's instructions. The concentration of total RNA was quantified by NanoDrop OneC Microvolume UV-Vis Spectrophotometer (Thermo). Reverse transcription was performed with SuperScript IV First-Strand Synthesis System (Thermo, 18091200) using 1ug total RNA according to manufacturer's instructions. qPCR was performed with FastStart Universal SYBR Green Master reagents (Sigma, 4913914001) and QuantStudio 5 Real-Time PCR System (Thermo). The results were analyzed with Design & Analysis Software (Thermo). The primer sequences were acquired from Origene (HP200471, HP200705, HP200090, HP225972).

Table 1. List of antibodies

Name	Company	Cat. #	Source	Application	ratio
AHR	Cell Signaling	83200	Rb	IB/IP	1:1000/1:50
p-AHR	Sino Biological	SBI200427-2	Rb	IB	1:500
□β-Actin	Cell Signaling	8457	Rb	IB	1:5000
DIO2	Proteintech	26513-1-AP	Rb	IB/IHC	1:1000/1:200
E-Cad	Cell Signaling	3195	Rb	IB	1:1000

FLAG-tagged	Sigma	F3165	Ms	IB	1:1000
GAPDH	Cell Signaling	5174	Rb	IB	1:10000
p-H3	Millipore	06-570	Rb	IB	1:1000
H3	Cell Signaling	4499	Rb	IB	1:1000
HA-tagged	Cell Signaling	3724	Rb	IB	1:1000
N-Cad	Cell Signaling	13116	Rb	IB	1:1000
PLK1	Millipore	05-844	Ms	IB/IP	1:1000/1:50
VIM	Cell Signaling	5741	Rb	IB	1:1000
HRP-linked goat anti-Rabbit IgG	Cell Signaling	7074	Gt	IB	1:3000
HRP-linked goat anti-Mouse IgG	Cell Signaling	7076	Gt	IB	1:3000
Normal Mouse IgG	Santa Cruz	sc-2025	Ms	IP	Match anti-PLK1 (IP)
Normal Rabbit IgG	Cell Signaling	2729	Rb	IP	Match anti-AHR (IP)

Table 2. List of chemicals

Name	Company	Cat. #	Solvent
Doxycycline (DOX)	MCE	HY-N0565	DMSO
Iopanoic acid (IOP)	MCE	HY-B1664	DMSO
Triiodothyronine (T3)	MCE	HY-A0070	DMSO
Tetraiodothyronine (T4)	MCE	HY-18341B	DMSO
Onvansertib (ONV)	MCE	HY-15828	DMSO
Nocodazole (NOC)	MCE	HY-13520	DMSO
CH-223191 (CH)	MCE	HY-12684	DMSO

Table 3. List of siRNAs and shRNAs

Name	Cat. #	Vector	Resistance	Targeting Region	Usage
siDIO2 #1	SASI_Hs01_00029989	N/A	N/A	NM_013989-1780	A549-SD
siDIO2 #2	SASI_Hs01_00029990	N/A	N/A	NM_013989-1811	A549-SD
siRNA-NC	SIC001	NA	N/A	NA	A549-SD
shDIO2 #1	TRCN0000084067	pLKO.1-puro	Puromycin	CDS	A549-SD
shDIO2 #2	TRCN0000294313	pLKO.1-puro	Puromycin	3'-UTR	A549-SD
pLKO.1-puro empty vector	SHC001	pLKO.1-puro	Puromycin	NA	A549-SD

References

1. Li, B. and C.N. Dewey, *RSEM: accurate transcript quantification from RNA-Seq data with or without a reference genome.* BMC Bioinformatics, 2011. **12**: p. 323.

2. Robinson, M.D., D.J. McCarthy, and G.K. Smyth, *edgeR: a Bioconductor package for differential expression analysis of digital gene expression data.* Bioinformatics, 2010. **26**(1): p. 139-40.

3. Wagner, G.P., K. Kin, and V.J. Lynch, *Measurement of mRNA abundance using RNA-seq data: RPKM measure is inconsistent among samples.* Theory Biosci, 2012. **131**(4): p. 281-5.

4. Mootha, V.K., et al., *PGC-1α-responsive genes involved in oxidative phosphorylation are coordinately downregulated in human diabetes.* Nature Genetics, 2003. **34**: p. 267.

5. Subramanian, A., et al., *Gene set enrichment analysis: a knowledge-based approach for interpreting genome-wide expression profiles.* Proc Natl Acad Sci U S A, 2005. **102**(43): p. 15545-50.

6. Liberzon, A., et al., *The Molecular Signatures Database (MSigDB) hallmark gene set collection.* Cell Syst, 2015. **1**(6): p. 417-425.

7. Gao, J., et al., *Integrative analysis of complex cancer genomics and clinical profiles using the cBioPortal.* Sci Signal, 2013. **6**(269): p. pl1.

8. Cerami, E., et al., *The cBio cancer genomics portal: an open platform for exploring multidimensional cancer genomics data.* Cancer Discov, 2012. **2**(5): p. 401-4.

9. *Comprehensive molecular profiling of lung adenocarcinoma.* Nature, 2014. **511**(7511): p. 543-50.

10. Jumper, J., et al., *Highly accurate protein structure prediction with AlphaFold.* Nature, 2021. **596**(7873): p. 583-589.

11. Margreitter, C., D. Petrov, and B. Zagrovic, *Vienna-PTM web server: a toolkit for MD simulations of protein post-translational modifications.* Nucleic Acids Res, 2013. **41**(Web Server issue): p. W422-6.

12. Reif, M.M., P.H. Hünenberger, and C. Oostenbrink, *New Interaction Parameters for Charged Amino Acid Side Chains in the GROMOS Force Field.* J Chem Theory Comput, 2012. **8**(10): p. 3705-23.

13. Abraham, M.J., et al., *GROMACS: High performance molecular simulations through multi-level parallelism from laptops to supercomputers.* SoftwareX, 2015. **1-2**: p. 19-25.

14. Darden, T., D. York, and L. Pedersen, *Particle mesh Ewald: An N·log(N) method for Ewald sums in large systems.* The Journal of Chemical Physics, 1993. **98**(12): p. 10089-10092.

15. Berendsen, H.J.C., et al., *Molecular dynamics with coupling to an external bath.* The Journal of Chemical Physics, 1984. **81**(8): p. 3684-3690.

16. Bussi, G., D. Donadio, and M. Parrinello, *Canonical sampling through velocity rescaling.* J Chem Phys, 2007. **126**(1): p. 014101.

17. Liu, X. and R.L. Erikson, *Polo-like kinase (Plk)1 depletion induces apoptosis in cancer cells.* Proc Natl Acad Sci U S A, 2003. **100**(10): p. 5789-94.

18. Li, C., et al., *GSTM2 is a key molecular determinant of resistance to SG-ARIs.* Oncogene, 2022. **41**(40): p. 4498-4511.

19. Barger, C.J., et al., *Pan-Cancer Analyses Reveal Genomic Features of FOXM1 Overexpression in Cancer.* Cancers (Basel), 2019. **11**(2).

20. Wiederschain, D., et al., *Single-vector inducible lentiviral RNAi system for oncology target validation.* Cell Cycle, 2009. **8**(3): p. 498-504.

21. Stewart, S.A., et al., *Lentivirus-delivered stable gene silencing by RNAi in primary cells.* Rna, 2003. **9**(4): p. 493-501.
