# Supplementary material for: Phosphorylation of AHR by PLK1 promotes metastasis of LUAD via DIO2-TH signaling
Source: PLoS Genet. 2023 Nov 21;19(11):e1011017. doi: 10.1371/journal.pgen.1011017 (PMC10662729; doi:10.1371/journal.pgen.1011017)

Immunoblot replicates for Fig. 1

B

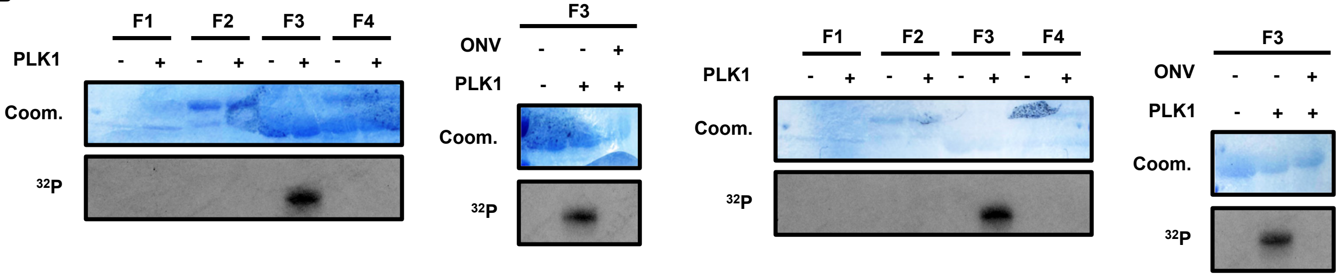

E

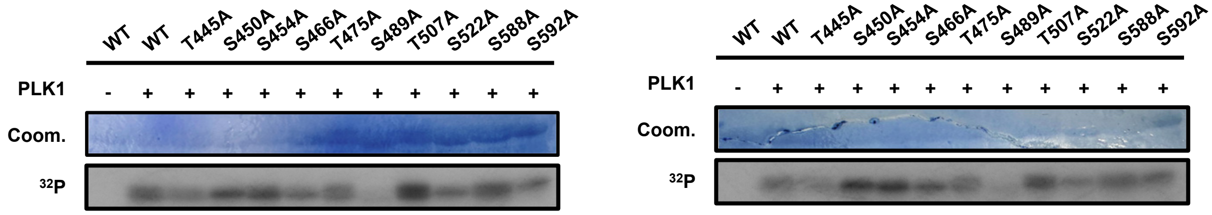

Immunoblot replicates for Fig. 2

C

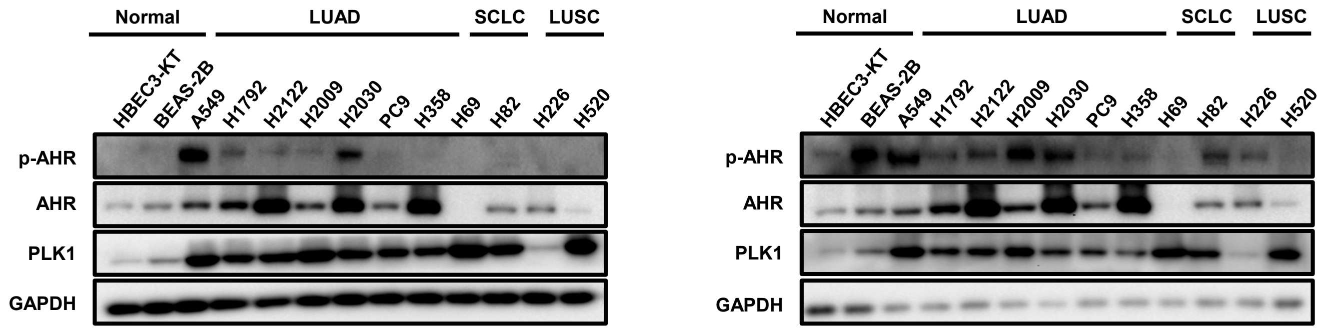

E

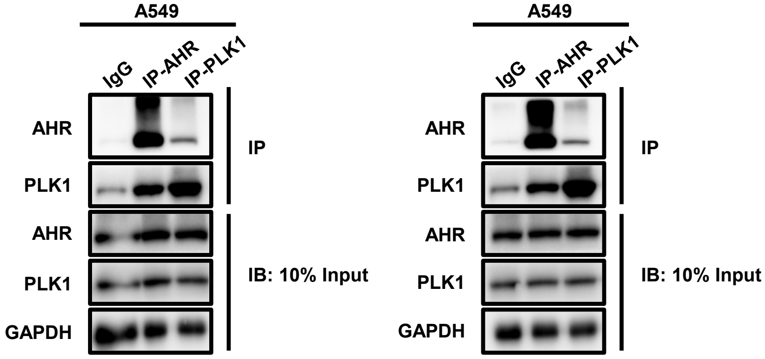

F

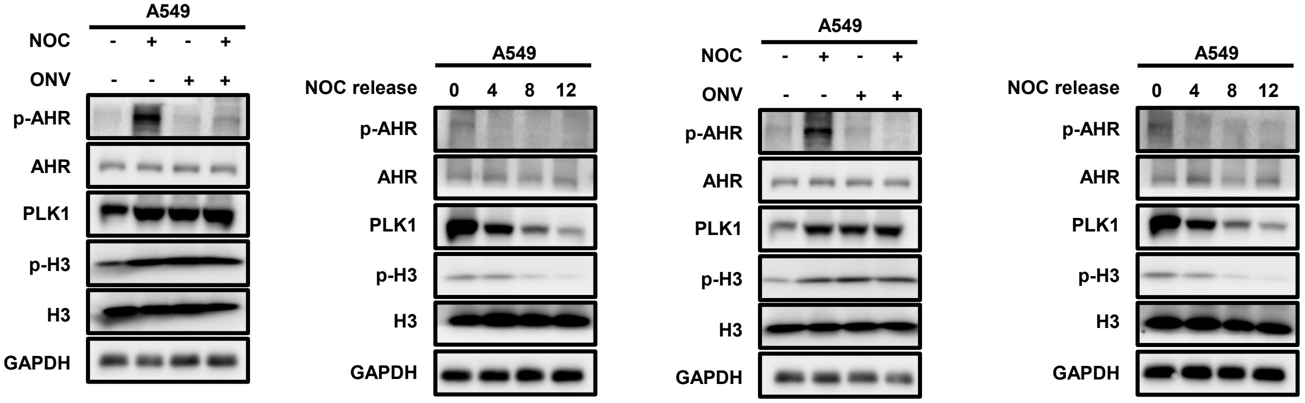

G

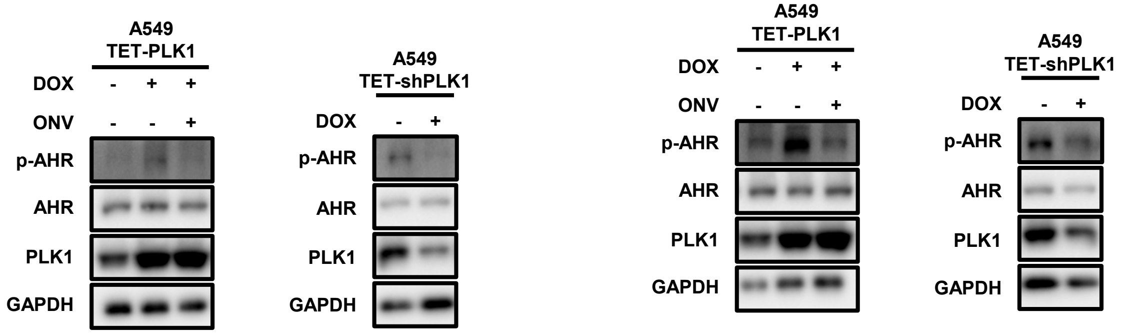

Immunoblot replicates for Fig. 3

B

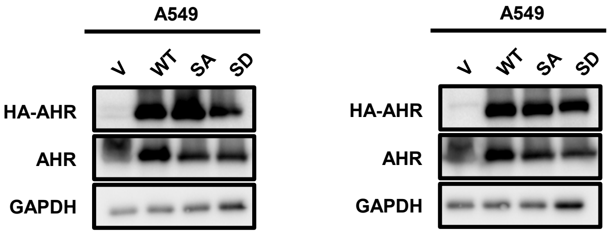

H

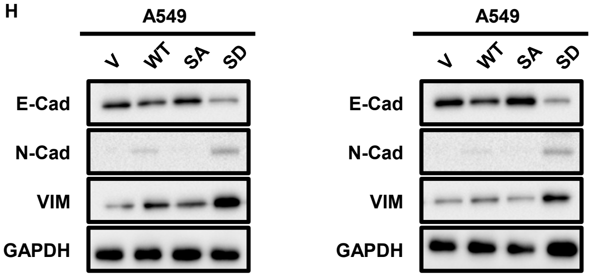

Immunoblot replicates for Fig. 5

G

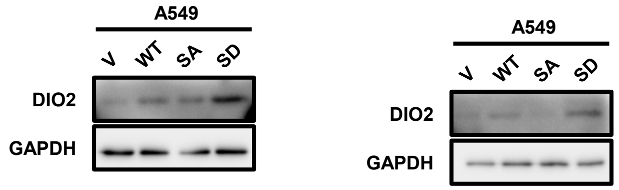

H

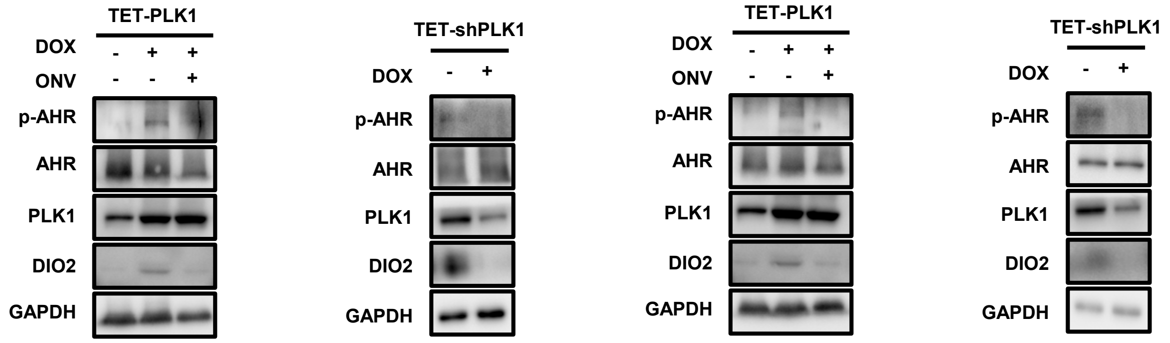

Immunoblot replicates for Fig. 6

B

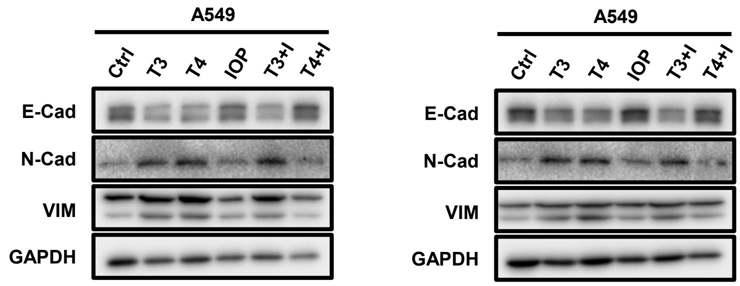

C

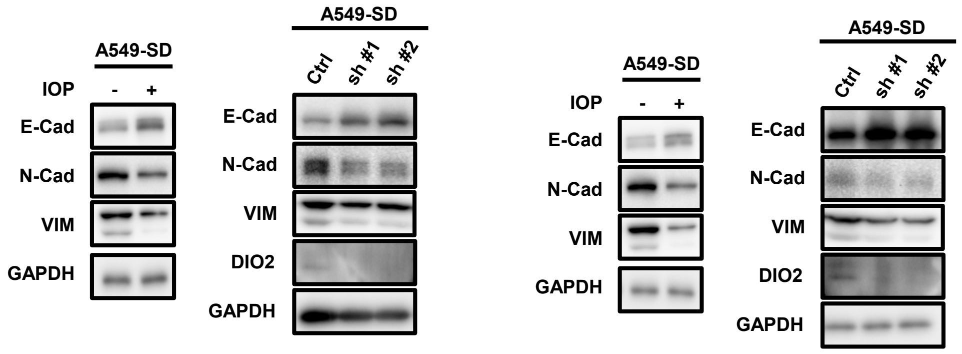

Immunoblot replicates for supplementary figures

S4A

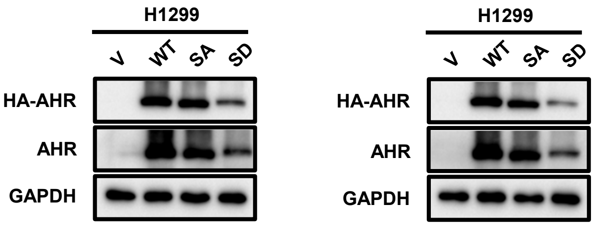

S9D

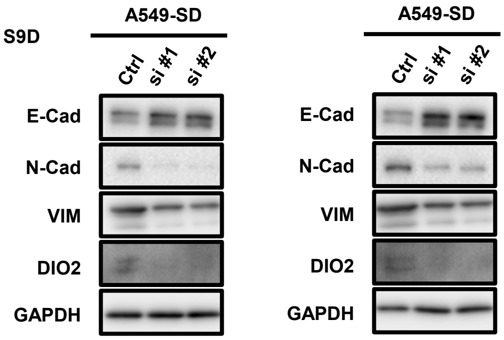

Supplement: S2 Appendix — (PDF) [file pgen.1011017.s018.pdf]
